# Fringe-Based Structured-Light 3D Reconstruction: Principles, Projection Technologies, and Deep Learning Integration

**DOI:** 10.3390/s25206296

**Published:** 2025-10-11

**Authors:** Zhongyuan Zhang, Hao Wang, Yiming Li, Zinan Li, Weihua Gui, Xiaohao Wang, Chaobo Zhang, Xiaojun Liang, Xinghui Li

**Affiliations:** 1 Shenzhen International Graduate School, Tsinghua University, Shenzhen 518000, China; zhongyua24@mails.tsinghua.edu.cn (Z.Z.); wangh23@mails.tsinghua.edu.cn (H.W.); lizn23@mails.tsinghua.edu.cn (Z.L.); wang.xiaohao@sz.tsinghua.edu.cn (X.W.); 2 Pengcheng Laboratory, Shenzhen 518000, China; lemonymtree@163.com (Y.L.); gwh@csu.edu.cn (W.G.); zhangchb@pcl.ac.cn (C.Z.); 3 School of Automation, Central South University, Changsha 410083, China

**Keywords:** fringe structured light, fringe projection profilometry, phase measuring deflectometry, deep learning, 3D measurement

## Abstract

Structured-light 3D reconstruction is an active measurement technique that extracts spatial geometric information of objects by projecting fringe patterns and analyzing their distortions. It has been widely applied in industrial inspection, cultural heritage digitization, virtual reality, and other related fields. This review presents a comprehensive analysis of mainstream fringe-based reconstruction methods, including Fringe Projection Profilometry (FPP) for diffuse surfaces and Phase Measuring Deflectometry (PMD) for specular surfaces. While existing reviews typically focus on individual techniques or specific applications, they often lack a systematic comparison between these two major approaches. In particular, the influence of different projection schemes such as Digital Light Processing (DLP) and MEMS scanning mirror–based laser scanning on system performance has not yet been fully clarified. To fill this gap, the review analyzes and compares FPP and PMD with respect to measurement principles, system implementation, calibration and modeling strategies, error control mechanisms, and integration with deep learning methods. Special focus is placed on the potential of MEMS projection technology in achieving lightweight and high-dynamic-range measurement scenarios, as well as the emerging role of deep learning in enhancing phase retrieval and 3D reconstruction accuracy. This review concludes by identifying key technical challenges and offering insights into future research directions in system modeling, intelligent reconstruction, and comprehensive performance evaluation.

## 1. Introduction

Three-dimensional reconstruction technology is a key approach for recovering the spatial structure of objects from images or sensor data, and it has been widely applied in various fields such as industrial inspection, medical imaging, cultural heritage digitization, and virtual reality [1,2,3,4,5]. Based on the method of acquiring depth information, 3D reconstruction can be categorized into passive and active approaches. Passive methods rely on natural illumination and image matching—typical examples include stereo vision and multi-view geometry. However, their reconstruction accuracy is often limited by factors such as texture richness and occlusions, making them unsuitable for high-precision measurement tasks [6,7,8,9,10]. In comparison, active 3D measurement techniques maintain high reconstruction accuracy even in regions with weak or absent texture features. By introducing an additional structured light source, they provide phase information to the measured area, thereby improving the accuracy and completeness of the 3D surface data. The laser triangulation method relies on the principle of triangulation rather than phase information for reconstruction [11,12]; however, due to its line-scanning nature, its speed is generally lower than that of area-based structured-light methods. The Time-of-Flight (TOF) method estimates depth information by measuring the travel time of laser pulses between the detector and the object, and it is typically applied in large-scale scenarios on the order of hundreds of meters [13,14].

Among various active techniques, structured light has emerged as a mainstream approach for high-precision 3D reconstruction at close range [15,16,17,18], owing to its high resolution, accuracy, and system flexibility [19,20,21]. It is widely applied in scenarios such as industrial surface inspection [22,23], facial recognition [24,25], and 3D modeling [26,27]. Most structured-light systems are based on phase encoding principles and can be broadly categorized into two representative methods: FPP and PMD. FPP is suitable for diffuse surfaces and reconstructs 3D shapes by projecting multiple phase-shifted fringe patterns and extracting their phase. In contrast, PMD is designed for specular or highly reflective surfaces, acquiring gradient information by analyzing the phase variations of reflected fringe patterns, from which the 3D structure is reconstructed [28,29,30]. Depending on the projection mechanism, FPP systems can be implemented in several ways, with the most common being DLP projectors and MEMS-based micromirror systems. DLP systems offer high pattern quality and fast refresh rates, making them the dominant solution. While DLP projectors have been extensively studied and widely applied in structured-light systems, discussions often focus on their optical design and depth-of-field characteristics. In comparison, micro-electro-mechanical systems (MEMS)-based projection has received relatively less attention, despite offering distinctive advantages. By generating patterns through laser scanning, MEMS projectors naturally enable large depth-of-field projection without the need for additional focusing optics. Moreover, their compactness and lightweight design make them well-suited for complex environments and mobile platforms [31,32]. In recent years, MEMS projection has attracted increasing attention as a promising direction for lightweight structured-light systems.

A number of scholars have conducted systematic reviews and studies focusing on key components of the structured-light 3D reconstruction pipeline. Tobias Möller provided an early overview of all-solid-state PMD range imaging, highlighting its feasibility, the 2005 “Hermes Award” commercial product, and key challenges such as background illumination and temperature variations that demand robust solutions [33]. Building upon these foundations, Xu et al. categorized and summarized the system architecture of PMD, analyzing critical issues such as measurement accuracy, system complexity, and calibration difficulty [34]. He et al. systematically compared three common temporal-phase unwrapping methods in FPP—namely, Temporal Filtering, Phase Coding, and Gray-Code—and evaluated their error characteristics and reconstruction performance under different system configurations [35]. Lv et al. optimized fringe orientation, pixel matching, and 3D reconstruction models from a theoretical perspective, proposing an FPP method that balances accuracy, efficiency, and implementation simplicity [36]. Bai et al. reviewed key techniques in full-field phase-based 3D measurement, including phase error compensation, high-speed image acquisition, and the application of deep learning in complex scenarios [37]. Kulkarni and Rastogi surveyed mainstream fringe denoising algorithms, comparing their performance in terms of phase accuracy and edge preservation [38]. In parallel, Liu et al. reviewed the progress of deep learning in fringe projection, summarizing representative methods, network structures, datasets, and application scenarios, and providing a structured overview of key technical advances and future research trends in this rapidly evolving domain [39].

Although multiple technical modules of structured-light 3D reconstruction systems have been extensively studied, most existing reviews focus on a single method or specific application, and a systematic comparison between the two mainstream approaches—FPP and PMD—is still lacking. In particular, there is no unified understanding of how different projection schemes, such as DLP and MEMS, affect system performance. To address this issue, this paper starts from the general paradigm of structured-light 3D reconstruction and provides a comprehensive review and comparison of FPP, PMD, and emerging MEMS technologies, focusing on key aspects such as measurement principles, system implementation, calibration and modeling, error control, and integration with deep learning. The paper emphasizes the differences in practical adaptability and the potential for integration among these approaches. Representative reviews and studies are summarized in Table 1.

**Figure 1:**
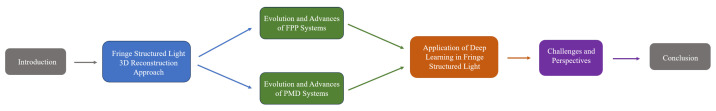
Flowchart of the article structure.

As illustrated in Figure 1, Section 1 introduces the research background and significance, while Section 2 starts from the general paradigm of fringe-structured-light 3D reconstruction, systematically presenting the principles of wrapped-phase extraction, phase unwrapping, and 3D shape recovery from phase, thereby laying the theoretical foundation for subsequent system evolution. Building on this paradigm, Section 3 focuses on the development of PMD systems, tracing their evolution from single-screen single-camera configurations to multi-screen direct PMD and multi-camera stereo PMD, gradually revealing their applicability and limitations in complex scenarios. In parallel, Section 4 shifts to FPP systems, analyzing the differences among mainstream projection technologies and examining calibration strategies and error modeling under MEMS-based projection, thereby highlighting challenges in accuracy and robustness. As traditional approaches increasingly reveal their shortcomings, Section 5 introduces the integration of deep learning into fringe-structured light, covering learning paradigms, network architecture innovations, supervision strategies, and input design, along with a discussion of evaluation metrics. Building upon these insights, Section 6 summarizes current challenges and outlines future research directions, including HDR imaging, extended depth of field, high-speed and real-time reconstruction, as well as the transferability and interpretability of deep learning methods. Finally, Section 7 concludes the paper by summarizing research progress and providing an outlook on future trends.

## 2. Fringe-Structured-Light 3D Reconstruction Approach

**Figure 2:**
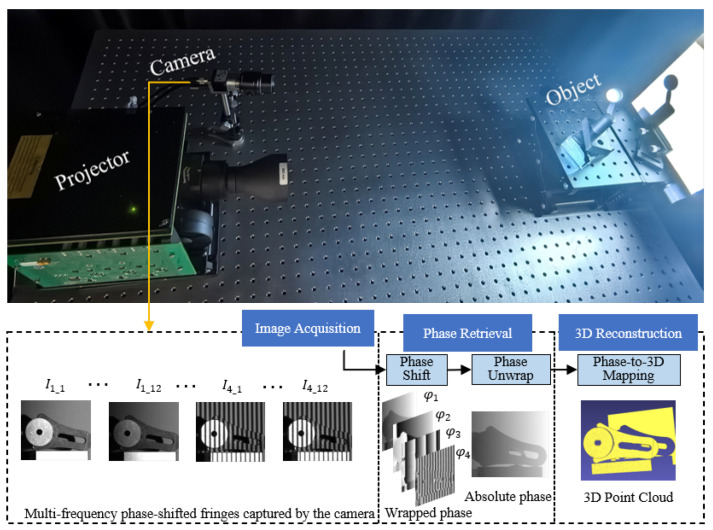
Typical experimental setup and workflow of FPP-based structured-light 3D reconstruction [44].

FPP and PMD are the two mainstream approaches in fringe-structured-light measurement, respectively, suited for 3D measurements of diffuse and specular surfaces. Although their system architectures differ, both methods fundamentally rely on projecting or displaying sinusoidal fringe patterns and utilizing the modulation effect imposed by the target object to recover 3D shape information [6,40,41]. As the fringe patterns undergo deformation on the object surface, their phase information directly reflects the spatial geometry of the surface. Therefore, a deterministic physical mapping exists between the phase and either depth (in FPP) or surface gradient (in PMD) [42,43]. With high-precision phase retrieval and phase unwrapping algorithms, FPP systems can construct depth maps, while PMD systems can reconstruct surface gradients and further recover the shape. Overall, the core pipeline of different fringe-structured-light 3D reconstruction methods can be abstracted as a physical sequence of “fringe modulation-phase retrieval-shape mapping.” The following sections will provide a step-by-step explanation of this reconstruction process. A representative experimental setup and workflow of FPP are illustrated in Figure 2, where the projector and camera are arranged to acquire deformed fringe patterns from the object. The subsequent processing pipeline includes phase retrieval, phase unwrapping, and mapping the recovered phase to 3D geometry, providing a concrete example of the general reconstruction process.

### 2.1. Wrapped-Phase Extraction

In the fringe analysis process, the primary task is to obtain the wrapped phase of the fringe pattern. Commonly used methods for wrapped-phase extraction include the phase-shifting method [45,46,47], wavelet transform method [48], and Fourier transform method [49]. Among these, the phase-shifting method has become the most widely adopted technique due to its high computational accuracy, strong robustness, and low sensitivity to environmental changes and noise [50]. In structured-light projection, sinusoidal fringe patterns are commonly adopted instead of binary patterns. The reason is that sinusoidal fringes provide smoother intensity transitions, leading to higher measurement accuracy and stronger robustness against noise and nonlinear response of the projector or camera. A typical implementation is the N-step phase-shifting method [51], where the generation of sinusoidal fringes can be described by the following equation:

(1) $$I\left(x,y\right)=I_{0}+I_{m}\cos\;\left(\frac{2\pi }{P}x\right)$$

where I(x,y) denotes the projected fringe intensity at pixel (x,y); $I_{0}$ is the minimum projection intensity, representing the lowest brightness level of the sinusoidal fringe; $I_{m}$ is the peak projection intensity, corresponding to the maximum brightness level; *P* is the fringe period; and *x* denotes the spatial coordinate along the fringe direction.

The projected fringe pattern from the projector can be described as follows:

(2) $$I_{n}\left(x,y\right)=I_{A}+I_{B}\cos\left[\varphi \left(x,y\right)-n\frac{2\pi }{N}\right]$$

where (x,y) denotes the coordinates of a pixel in the 2D image; $I_{n}\left(x,y\right)$ represents the intensity value at that pixel, i.e., the brightness or grayscale value of the image; $I_{A}$ is the background intensity, which includes ambient light and the unmodulated portion of the signal; $I_{B}$ denotes the modulated intensity, which is related to the reflectivity of the object’s surface; n=0,1,2,…,N−1 is the number of phase shifts; and φ(x,y) is the phase at the pixel to be retrieved. According to the least squares method, the wrapped phase of the object can be calculated as follows:

(3) $$\varphi \left(x,y\right)=\arctan\left(\frac{\sum_{n=0}^{N-1}I_{n}\left(x,y\right)\sin\left(\frac{2\pi n}{N}\right)}{\sum_{n=0}^{N-1}I_{n}\left(x,y\right)\cos\left(\frac{2\pi n}{N}\right)}\right)$$

The Fourier transform method is a single-frame phase extraction technique based on frequency-domain analysis. In this approach, a sinusoidal fringe pattern with a specific frequency is projected onto the object. The captured image is then transformed into the frequency domain, where filtering operations are applied to isolate the fundamental frequency component. An inverse Fourier transform is subsequently performed to recover the phase information of the fringe pattern. The primary advantage of this method lies in its ability to compute the phase from just a single image, making it well-suited for dynamic objects or real-time measurement scenarios.

According to Euler’s formula, the fringe image can be expressed as follows:

(4) $$\begin{array}{cc} I(x,y) & =I_{A}+I_{B}\cos\left[\varphi \left(x,y\right)+2\pi f_{0}x\right]\\ \; & =I_{A}+I_{c}+I_{c}^{\prime }\\ \; & =I_{A}+\frac{1}{2}I_{B}e^{i(\varphi \left(x,y\right)+2\pi f_{0}x)}+\frac{1}{2}I_{B}e^{-i(\varphi \left(x,y\right)+2\pi f_{0}x)} \end{array}$$

Applying the Fourier transform to Equation (3) along the x-direction yields the following:

(5) $$I\left(f\right)=I_{A}\left(f\right)+I_{c}\left(f-f_{0}\right)+I_{c}^{\prime }\left(f+f_{0}\right)\;$$

The Fourier spectrum of the fringe image primarily consists of three frequency bands: the −1st order $I_{c}\left(f-f_{0}\right)$, the 0th order $I_{A}\left(f\right)$, and the +1st order conjugate component $I_{c}^{\prime }\left(f+f_{0}\right)$. Among these, the 0th order component represents the zero-frequency term and reflects the background intensity distribution, while the ±1st order components contain the essential phase information of the fringe pattern.

In practical applications, a band-pass filter is typically applied to retain the +1st order component and suppress the other frequency components, thereby enhancing the accuracy of phase extraction. The retained component is then subjected to an inverse Fourier transform, yielding

(6) $$I_{c}=\frac{1}{2}I_{B}\left(\cos\left(\varphi \left(x,y\right)+2\pi f_{0}x\right)+i\sin\left(\varphi \left(x,y\right)+2\pi f_{0}x\right)\right)$$

The real and imaginary parts of the Fourier spectrum of the fringe image can be expressed as follows:

(7) Re{Ic}=12IBcos(φ(x,y)+2πf0x)

(8) Im{Ic}=12IBsin(φ(x,y)+2πf0x)

Therefore, the wrapped phase of the object can be expressed as follows:

(9) φ(x,y)=arctanIm{Ic}Re{Ic}

It is important to note that the obtained φ(x,y) is the wrapped phase, with values that are confined within the range (−π,π] and exhibit periodic discontinuities. Therefore, a subsequent phase unwrapping step is required to eliminate these discontinuities and recover the true absolute phase, which is essential for accurate 3D reconstruction.

### 2.2. Phase Unwrapping Algorithms

According to the dimensional source of information utilized during the phase unwrapping process, phase unwrapping methods in structured-light 3D reconstruction can be broadly categorized into temporal-phase unwrapping (TPU) and spatial-phase unwrapping (SPU).

#### 2.2.1. Temporal-Phase Unwrapping

TPU refers to a class of methods that project multiple fringe patterns with different frequencies or encodings, and compute the absolute phase independently for each pixel based on the temporal variation in grayscale intensity. These methods do not rely on spatial continuity of the phase map, making them highly robust for surfaces with steep variations, discontinuities, or occlusions [52]. Depending on the type of modulation encoding used, TPU methods can be further classified into the following: Gray-code Phase Unwrapping, Multi-frequency Phase Unwrapping, Multi-wavelength Phase Unwrapping.

**Figure 3:**
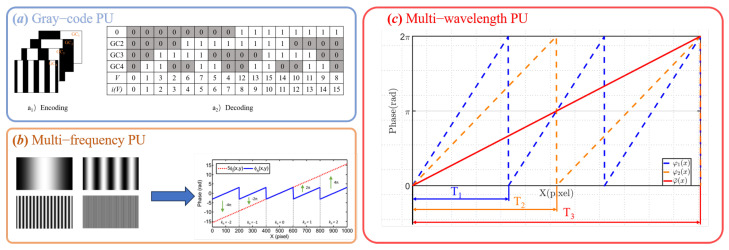
Illustration of three common phase unwrapping (TPU) methods. (**a**) Gray-code PU showing the binary-to-decimal decoding process. (**b**) Multi-frequency PU using different fringe periods for coarse-to-fine unwrapping. (**c**) Multi-wavelength PU leveraging synthetic wavelengths to extend the unwrapping range.

Gray-code Phase Unwrapping is a typical temporal-phase unwrapping method that combines structured encoding projection with the phase-shifting technique. It is widely used for absolute phase reconstruction tasks. The fundamental principle is as follows: a set of Gray-code patterns is first projected to encode the fringe periods pixel by pixel, allowing for the precise determination of each pixel’s fringe order. Subsequently, a set of sinusoidal phase-shifted fringe patterns is projected, from which the wrapped phase is extracted using a phase-shifting algorithm [53]. By integrating the encoded fringe order from the Gray-code and the wrapped phase from the phase-shifting method, the wrapped phase within the interval $\left(-\pi ,\pi \right]$ can be converted into a globally continuous absolute phase, enabling accurate 3D shape reconstruction. The encoding and decoding process is illustrated in Figure 3a.

Multi-frequency Phase Unwrapping is a representative temporal-phase unwrapping method. As illustrated in Figure 3b, this method utilizes the phase information obtained from low-frequency fringe patterns to assist in unwrapping the wrapped phase of high-frequency fringe patterns, thereby achieving a balance between high measurement accuracy and a large measurement range. Typically, this method involves projecting two or more sets of sinusoidal fringe patterns with different spatial frequencies and extracting the wrapped phase from each set independently [40].

(10) $$\left\{\begin{array}{cc} \Phi_{h}\left(x,y\right) & =\varphi_{h}\left(x,y\right)+2\pi k_{h}\left(x,y\right)\\ \Phi_{l}\left(x,y\right) & =\varphi_{l}\left(x,y\right)+2\pi k_{l}\left(x,y\right)\\ \Phi_{h}\left(x,y\right) & =\frac{f_{h}}{f_{l}}\Phi_{l}\left(x,y\right) \end{array}\right.$$

where $\Phi_{h}\left(x,y\right)$ and $\Phi_{l}\left(x,y\right)$ represent the unwrapped absolute phases of the high- and low-frequency fringes, respectively; $\varphi_{h}\left(x,y\right)$ and $\varphi_{l}\left(x,y\right)$ denote the wrapped phases extracted from the high- and low-frequency fringe patterns using the phase-shifting method; $k_{h}$ and $k_{l}$ are the fringe orders of the high- and low-frequency patterns, respectively; and $f_{h}$ and $f_{l}$ are the corresponding spatial frequencies of the projected fringe patterns.

To further resolve the fringe order $k_{h}\left(x,y\right)$, Equation (10) provides a rounding-based formulation that exploits the relationship between the high- and low-frequency wrapped phases. Specifically, the difference between the scaled low-frequency phase $\frac{f_{h}}{f_{l}}\Phi_{l}\left(x,y\right)$ and the high-frequency wrapped phase $\varphi_{h}\left(x,y\right)$ is normalized by 2π and then rounded to the nearest integer. This process effectively determines the correct fringe order by constraining the phase discrepancy within a 2π range, thereby enabling the reliable recovery of the absolute high-frequency phase.

(11) $$k_{h}\left(x,y\right)=\mathrm{Round}\left(\frac{\frac{f_{h}}{f_{l}}\Phi_{l}\left(x,y\right)-\varphi_{h}\left(x,y\right)}{2\pi }\right)$$

Once the fringe order $k_{h}$ is determined, the absolute phase can be progressively recovered across different frequencies.

Multi-wavelength phase Unwrapping is a temporal technique that leverages the principle of beat frequency. As illustrated in Figure 3c, its core idea is to project multiple sets of sinusoidal fringe patterns with closely spaced spatial frequencies (or equivalently, wavelengths) to synthesize a phase map with a significantly extended equivalent wavelength. This synthetic phase greatly increases the unambiguous measurement range and effectively mitigates phase ambiguity, thereby improving the robustness and accuracy of the final reconstruction.Typically, two sets of fringe patterns with closely spaced frequencies are used, denoted by spatial frequencies $f_{1}$ and $f_{2}$. The resulting synthetic phase map $\varphi_{eq}\left(x,y\right)$ and equivalent wavelength $\lambda_{eq}$ can be expressed as follows:

(12) $$\varphi_{eq}\left(x,y\right)=\varphi_{2}\left(x,y\right)-\varphi_{1}\left(x,y\right)$$

(13) $$\lambda_{eq}=\frac{1}{f_{eq}}=\frac{\lambda_{1}\lambda_{2}}{\lambda_{1}-\lambda_{2}}$$

Therefore, the fringe order $k_{2}$ can be expressed as follows:

(14) $$k_{2}\left(x,y\right)=\mathrm{Round}\left(\frac{\frac{\lambda_{eq}}{\lambda_{2}}\varphi_{eq}\left(x,y\right)-\varphi_{2}\left(x,y\right)}{2\pi }\right)$$

#### 2.2.2. Spatial-Phase Unwrapping

Unlike temporal-phase unwrapping, spatial-phase unwrapping techniques utilize phase information from neighboring pixels in space. By comparing phase differences between adjacent pixels, the method progressively removes the periodic discontinuities in the wrapped phase and recovers the true surface phase of the object. However, phase unwrapping errors in this approach tend to propagate from high-noise regions to low-noise areas and beyond. The computational strategies for spatial-phase unwrapping are generally divided into two categories: path-following local methods and path-independent global methods [54]. Among them, quality-guided unwrapping and branch-cut algorithms are representative local methods, while unweighted and weighted least-squares methods belong to the global category. Global phase unwrapping methods are typically based on the least-squares principle, which transforms the phase unwrapping problem into algebraic equations or matrix solutions to obtain a globally optimal result [55,56,57,58,59]. The basic idea is to convert the measured phase gradient field into a system of linear equations and recover the unwrapped phase through least-squares solutions (e.g., QR decomposition, i.e., orthogonal–triangular decomposition, or algebraic number theory methods). Although such methods are theoretically well-supported by algebraic and statistical tools, in practice, they tend to be sensitive to noise, less accurate in the presence of occlusions or fringe discontinuities, and computationally demanding, making them unsuitable for real-time applications. In contrast, local methods demonstrate greater robustness in handling noise, discontinuities, and complex surfaces, and thus remain the mainstream approaches in current research and applications.

**Figure 4:**
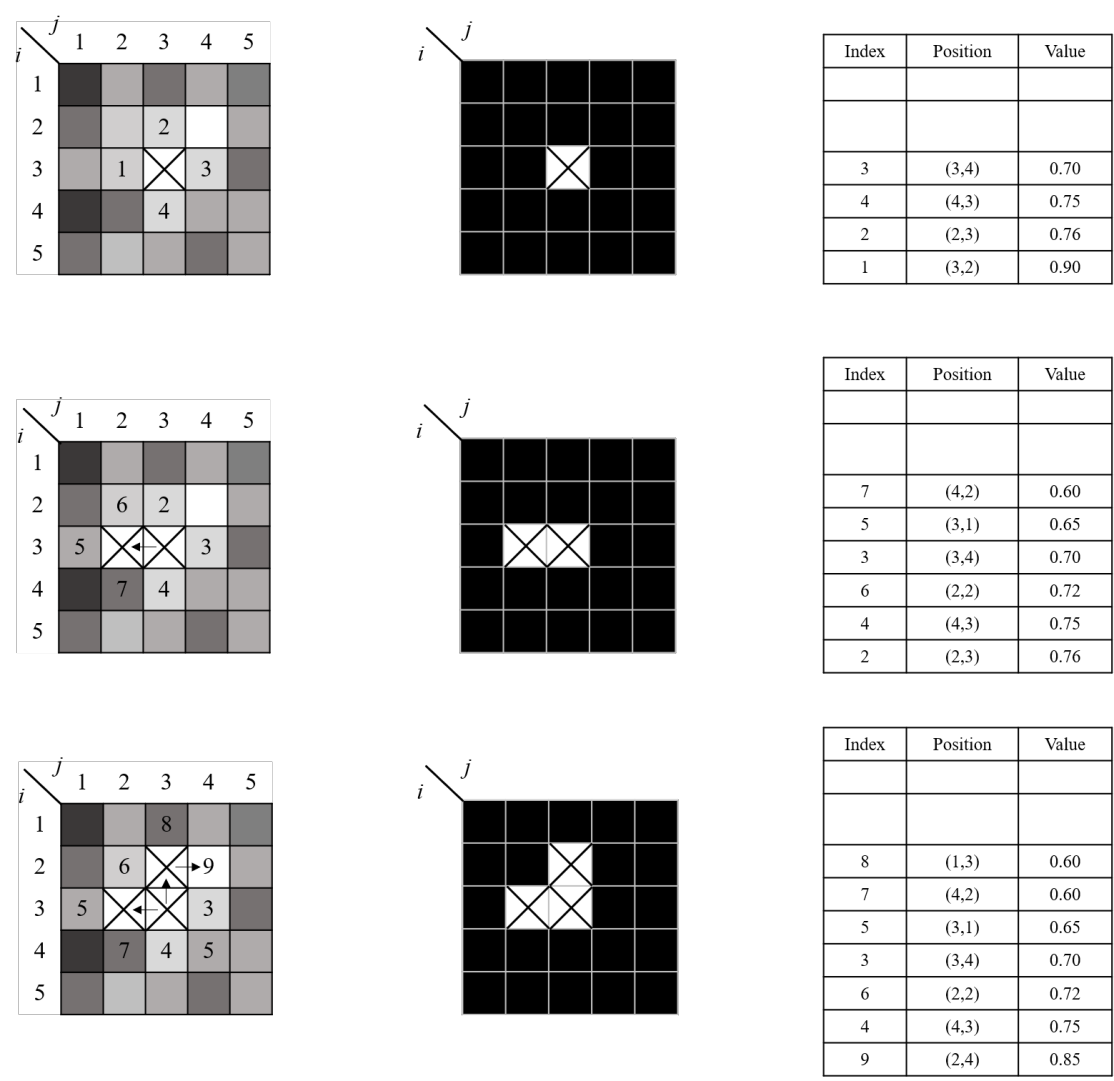
Schematic diagram of phase unwrapping based on directed parallel mapping [61].

Quality-Guided Phase Unwrapping has been widely studied due to its efficiency and speed [51,60]. This method evaluates the quality of the wrapped phase using a quality map, and applies a flood-fill algorithm to initiate unwrapping from high-quality regions. This strategy effectively limits the propagation of unwrapping errors into low-quality areas, thereby enhancing both accuracy and stability. Su et al. proposed a reliability-guided phase unwrapping method based on parameter mapping, in which one or more parameters—such as modulation of the fringe pattern, spatial frequency, phase differences between neighboring pixels, and signal-to-noise ratio—are used to construct a parameter map. The phase unwrapping path is then guided by the high-reliability regions of this map. As illustrated in Figure 4, this approach effectively confines phase unwrapping errors to localized areas and demonstrates strong robustness [61].

Branch-Cut Phase Unwrapping, also known as the Goldstein algorithm, was first proposed by Goldstein in 1988 [62], and is a commonly used path-dependent phase unwrapping algorithm. The main steps include the following: (1) identifying and labeling the polarity of phase residues; (2) constructing branch cuts to connect all residues and ensuring that the sum of the polarity values on each branch cut is zero; (3) bypassing the branch cuts during the unwrapping process and using the phase information from neighboring unwrapped pixels to unwrap the residues. Compared with quality-guided phase unwrapping, the branch-cut method offers stronger noise resistance. By constructing branch cuts and preventing error propagation, it effectively reduces the impact of noise on phase unwrapping.

However, the branch-cut method also has some limitations. In regions where phase residues are densely distributed, incorrect branch cuts may be generated, or the constructed branch cuts may not be globally shortest, which could lead to unwrapping errors. In addition, branch cuts may form closed loops, resulting in the “island effect,” which further aggravates local error accumulation. Therefore, the performance of the branch-cut method is highly dependent on the placement of cuts. If the noise level is high, significant unwrapping errors may occur. To address these problems, subsequent research has introduced several improvements to the Goldstein algorithm. For example, Huntley proposed placing artificial barriers or using independent unwrapping paths to avoid noise propagation and obtain unique and accurate phase unwrapping results [63]. Zheng introduced a random search-based method for locating branch cuts, which improves computational speed and solves the inaccuracy issue of branch cut construction in the Goldstein algorithm [64]. Gdeisat et al. proposed increasing the number of residues in the wrapped-phase map to improve unwrapping accuracy, but this method is computationally intensive and time-consuming [65]. To address this issue, Du et al. proposed a simplified algorithm that significantly speeds up computation, reducing processing time by more than 50% and effectively improving measurement efficiency [66].

### 2.3. 3D Shape Reconstruction from Phase

The recovery of 3D surface shape relies on the mapping relationship between phase and spatial geometry. In general, the phase information reflects the geometric modulation of fringe patterns on the surface of the measured object, and the degree of modulation depends on the optical path variation caused by the surface geometry. To reconstruct the 3D coordinates from the phase, PMD and FPP techniques each establish distinct geometric mapping models.

#### 2.3.1. 3D Shape Recovery in PMD

**Figure 5:**
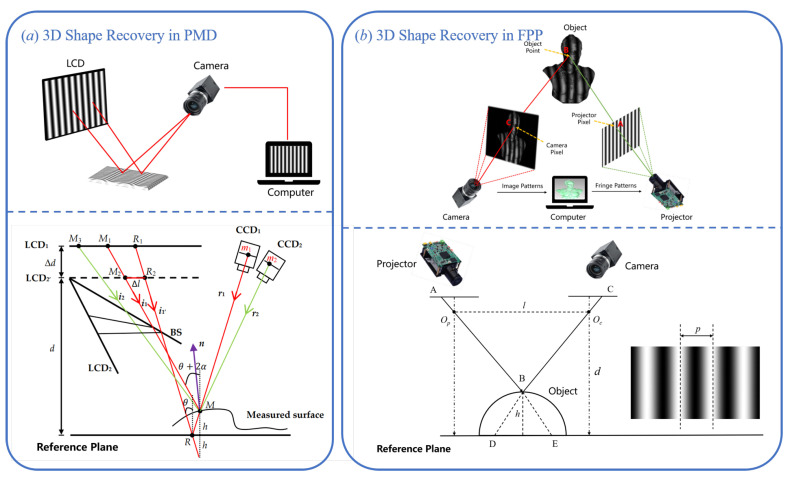
Comparison of 3D shape recovery methods. (**a**) PMD reconstructs surface gradients, which can be further integrated into object shape [37]. (**b**) FPP directly recovers depth maps via phase-based reconstruction.

PMD is an optical measurement technique specifically designed for 3D reconstruction of specular or highly reflective surfaces. As shown in the top part of Figure 5a, a typical PMD system consists of a liquid crystal display (LCD), a camera, and a computer. The computer generates sinusoidal fringe patterns and displays them on the LCD. These patterns are reflected by the mirror-like surface of the object and then captured by the camera. Because the specular surface geometrically modulates the fringe pattern, the captured image contains phase distortion information caused by variations in the surface normal [67,68]. After extracting the wrapped phase from the captured fringe images using techniques such as phase-shifting, the system uses a geometric model and calibration parameters to convert the phase information into the surface gradient data of the object [69]. Since the phase is proportional to the deflection angle of the surface normal vector, PMD essentially measures a gradient field that reflects the surface slope. To reconstruct the full 3D shape of the object, this gradient field must be numerically integrated over the 2D image plane to recover the relative height at each pixel, thereby producing the complete 3D surface profile [70].

In recent years, researchers have proposed a Direct Phase-Measuring Deflectometry (DPMD) system based on a dual-LCD and dual-camera setup. This architecture is designed to bypass the complex gradient integration process required in traditional PMD, enabling direct height reconstruction of specular objects [71,72]. As shown in the bottom part of Figure 5a, this method captures sinusoidal fringe patterns reflected from both a reference plane and the measured specular surface, using two LCD screens and two cameras. Each camera simultaneously acquires the distorted fringe images along two different optical paths, thereby recording the phase variations corresponding to these paths. When the fringe patterns are reflected by the object and the reference plane, the images captured by the cameras contain the phase difference between the two reflection paths. Through system calibration, this phase difference can be directly mapped to height differences on the object surface, effectively eliminating the gradient integration step required in traditional PMD. The modeling principles and technical details of this method will be further discussed in Section 3.2.

#### 2.3.2. 3D Shape Recovery in FPP

FPP is an active optical 3D measurement technique based on phase encoding, widely used for measuring diffuse reflective surfaces. It has attracted significant attention due to its simple structure, high accuracy, and broad applicability. The core principle of FPP is to project periodic sinusoidal fringe patterns onto the surface of the measured object under known geometric relationships between the projection direction and the camera’s viewing angle. The fringe patterns are distorted by the surface geometry of the object. After being captured by the camera, the 3D shape of the surface can be reconstructed through phase decoding.

As illustrated in the top part of Figure 5b, a typical FPP system consists of a projector, the object being measured, and a camera. A geometric imaging model is established among these three components through spatial calibration. The computer controls the projector to display a sequence of phase-shifted sinusoidal fringe patterns onto the object’s surface, while the camera synchronously captures the deformed fringe images. According to the procedure described in Section 2.2, the absolute phase of the object can be retrieved. Once phase unwrapping is completed and phase discontinuities are removed, the phase information becomes spatially continuous [73]. After obtaining the absolute phase, the system must convert the phase values into the actual 3D coordinates of the object surface using a calibration model. The core task of this model is to establish a mapping between the absolute phase and the spatial geometric information. Depending on the modeling approach, these calibration models are generally classified into two categories: the phase-height model and the triangulation model [74].

The phase-height model is a method that establishes a direct functional relationship between phase and height using multiple reference planes with known elevations. It is well-suited for scenarios where the object is located near the reference plane and the surface variation is relatively smooth. Common phase-height models can be generally classified into three categories, linear models [75], inverse linear models [76], and polynomial models [77,78].

A classic phase-height model is illustrated in the bottom part of Figure 5b, where $\Delta \Phi_{DE}\left(x,y\right)$ denotes the phase difference between the object and the reference plane, $O_{p}$, $O_{c}$ represent the optical centers of the projector and camera, respectively, *l* denotes the baseline distance between them, *d* is the vertical distance between the camera and the reference plane, and *p* is the width of a projected stripe on reference plane. Let *B* be a point on the surface of the measured object, and let *h* denote the height of point *B* relative to the reference plane. According to the principle of triangulation, the height *h* of point *B* can be expressed as [79].

(15) $$h=\frac{\Delta \Phi_{DE}\cdot p\cdot d}{\Delta \Phi_{DE}\cdot p+2\pi l}$$

where *p*, *l*, and *d* are the parameters that need to be calibrated in the phase-height model.

If the measurement system satisfies $l\gg \bar{DE}$, and the actual height distribution of the object is not uniform, then according to Equation (14), the linear phase-height relationship can be expressed as follows:

(16) $$h\left(x,y\right)=\frac{\Delta \Phi_{DE}\left(x,y\right)\cdot p\left(x,y\right)\cdot d}{2\pi l}=k\left(x,y\right)\Delta \Phi_{DE}\left(x,y\right)$$

where k(x,y) is a proportional coefficient to be calibrated, which can be obtained through least-squares fitting using known heights from a set of reference planes. To improve modeling accuracy, phase values are typically collected at multiple height levels, and pixel-wise fitting is performed to determine k(x,y), thereby yielding more accurate local reconstruction results. The linear phase-height model is simple to implement and computationally efficient, making it suitable for fast measurement tasks. However, when the system’s structural parameters do not satisfy the approximation condition ($l\gg \bar{DE}$), the accuracy of the linear model degrades significantly.

To relax the strict geometric assumptions required by the traditional linear model, researchers have proposed the inverse linear phase-height model. This model introduces a reciprocal relationship between phase and height, establishing a linear mapping between the reciprocal of height and the reciprocal of the phase difference.

(17) $$\frac{1}{h(x,y)}=a\left(x,y\right)+b\left(x,y\right)\cdot \frac{1}{\Delta \Phi_{DE}\left(x,y\right)}$$

where a(x,y) and b(x,y) are the calibration coefficients to be determined for each pixel. This model allows for more flexible configurations of the camera and projector, requiring only a shared field of view for measurement, without the need for strict coplanarity between the projection path and the reference plane. By applying least-squares fitting using multiple reference planes with known heights, the coefficients a(x,y) and b(x,y) can be efficiently determined, thus completing the system calibration. It is worth noting that Equation (16) can be rearranged as follows:

(18) $$\Delta \Phi_{DE}\left(x,y\right)=h\left(x,y\right)\Delta \phi_{DE}\left(x,y\right)a\left(x,y\right)+h\left(x,y\right)b\left(x,y\right)$$

Although the two equations mentioned above appear to be different forms of the same expression, in practical applications, Equation (16) is more susceptible to noise, which can lead to significant error amplification in regions with large object height, indicating its dependency on object height [77,80]. In contrast, Equation (17) demonstrates stronger robustness against noise.

By further rearranging Equation (18), we obtain the following:

(19) $$h\left(x,y\right)=\frac{\Delta \Phi_{DE}\left(x,y\right)}{a\left(x,y\right)\Delta \phi_{DE}\left(x,y\right)+b\left(x,y\right)}$$

This equation reflects the nonlinear relationship between the phase difference $\Delta \Phi_{DE}\left(x,y\right)$ and the object height h(x,y) [77]. However, the nonlinear fitting process depends heavily on the initial values of a(x,y) and b(x,y), which can affect the overall calibration accuracy and system stability. To address this issue, some researchers have proposed using polynomial fitting to model the nonlinear relationship more flexibly [78]. In this case, the height h(x,y) can be expressed as a polynomial function of the phase difference:

(20) $$h\left(x,y\right)=\sum_{i=0}^{n}a_{i}\left(x,y\right){\left[\Delta \Phi_{DE}\left(x,y\right)\right]}^{i}$$

It is worth noting that although increasing the polynomial order can improve the accuracy of fitting the nonlinear relationship, an excessively high order may lead to Runge’s phenomenon [81]. Therefore, the degree of the polynomial should be carefully selected to balance fitting accuracy and model stability.

In the phase-height models described above, the system typically does not perform geometric modeling or calibration of the camera and projector. Instead, it fits a functional relationship between phase and height through empirical calibration. In contrast, the triangulation model requires precise calibration of both the camera and the projector in order to recover the 3D coordinates of the object’s surface using the principle of triangulation. A projector can be treated as an inverse camera, and its geometric parameters can be calibrated using methods similar to those used for cameras. However, unlike a camera, the projector cannot directly form an image. Therefore, it requires the assistance of a reflective surface—either the measured object or a reference plane—to reflect fringe patterns, and relies on phase encoding to establish the correspondence between projector pixels and camera pixels. In this process, phase information plays a key role in pixel matching.

**Figure 6:**
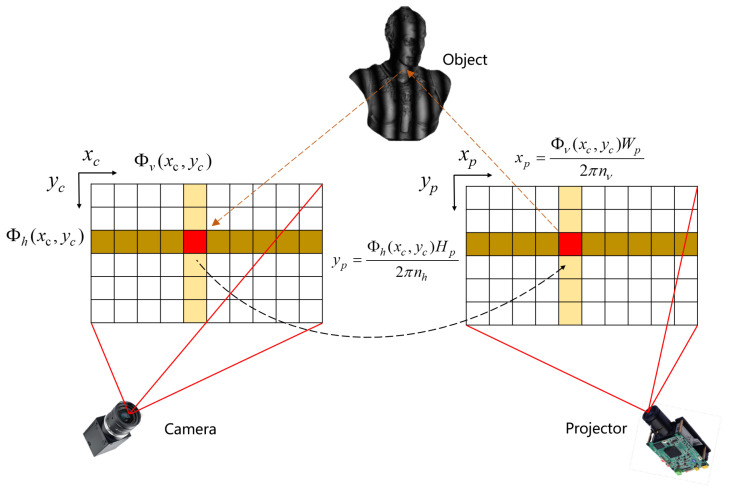
Illustration of how the projector observes the measurement point with the aid of cameras [74].

As illustrated in Figure 6, projector calibration typically involves projecting vertical and horizontal fringe patterns. Using phase-shifting and temporal-phase unwrapping algorithms, the absolute phase in the vertical direction, $\Phi_{v}\left(x_{c},y_{c}\right)$, and the absolute phase in the horizontal direction, $\Phi_{h}\left(x_{c},y_{c}\right)$, can be obtained for each pixel.

Assuming the projector resolution is $H_{p}\times W_{p}$, with $n_{v}$ vertical fringes and $n_{h}$ horizontal fringes in the projected patterns, a camera pixel at ${\left(x_{c},y_{c}\right)}^{T}$ corresponds to a point ${\left(x_{p},y_{p}\right)}^{T}$ on the projector pixel plane. The coordinates can be computed as follows:

(21) $$\begin{array}{cc} x_{p} & =\frac{\Phi_{v}\left(x_{c},y_{c}\right)W_{p}}{2\pi n_{v}} \end{array}$$

(22) $$\begin{array}{cc} y_{p} & =\frac{\Phi_{h}\left(x_{c},y_{c}\right)H_{p}}{2\pi n_{h}} \end{array}$$

The projector can then be calibrated by following the same procedure as camera calibration [82].

## 3. Evolution and Advances of PMD Systems

PMD is a 3D measurement technique based on the laws of optical reflection, specifically designed for reconstructing the 3D shape of highly smooth, specular surfaces. The fundamental idea is to project sinusoidal phase-shifted fringe patterns onto a display screen and to use a camera to capture the modulated images of these patterns reflected from the object’s surface. Phase information is then extracted from the captured images to infer the surface normals or height distribution of the object. PMD is essentially a reflection-based structured-light method, and it is closely related in principle to Moiré deflectometry [83,84,85,86] while offering stronger advantages in terms of measurement accuracy, dynamic range, and system adaptability [87,88,89,90,91,92]. Depending on the system configuration, existing PMD systems can be categorized into three types: Single-screen and single-camera PMD systems, Multi-screen direct PMD systems, and Multi-camera stereo PMD systems [34].

### 3.1. Single-Screen and Single-Camera Systems

Among all PMD configurations, the single-screen and single-camera system has been widely adopted in both early and contemporary research on specular surface 3D measurement, due to its compact structure and minimal construction complexity [93]. This system typically consists of an LCD, a camera, and a computer. The computer controls the screen to project a sequence of sinusoidal fringe patterns onto the surface of the specular object. The camera, positioned in the reflection direction, captures the modulated fringe patterns. Through phase extraction and surface reconstruction algorithms, the 3D geometry of the surface is recovered.

**Figure 7:**
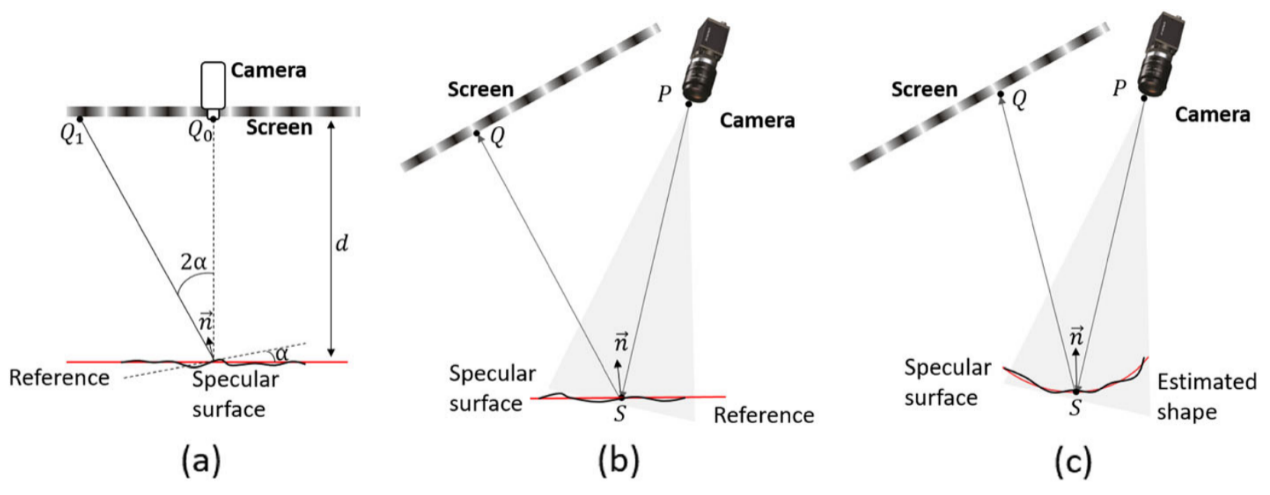
Illustration of three typical phase-to-height mapping models. (**a**) Paraxial approximation model. (**b**) Planar reference-based model. (**c**) Shape estimation and reprojection model [34].

As shown in Figure 7, typical single-screen single-camera PMD systems can be modeled using three different approaches, paraxial approximation model, reference-plane-based model, and surface estimation and reprojection model. The paraxial approximation model assumes small incidence and reflection angles, making it well-suited for standard specular surface measurement tasks but less accurate for large-angle scenarios. In contrast, the planar reference-based model introduces a physical reference plane to extend the applicable range, though its accuracy depends on precise calibration. The reprojection model further relaxes the small-angle constraint by incorporating full geometric relationships, thereby achieving higher accuracy in complex or large-angle measurement conditions.

The paraxial approximation model has been widely used in standard specular surface measurement tasks. This approach typically assumes that the angle between the reflected fringe direction and the surface normal is small, allowing a simplified phase-to-height mapping to be established. Based on this assumption, Häusler et al. proposed a compact single-screen single-camera PMD system suitable for objects with relatively small surface variations [94]. Later, Liu et al. further optimized the geometry of this model to maintain high measurement accuracy even when measuring mildly curved surfaces [95]. Due to its mathematical simplicity and ease of implementation, the paraxial model has been adopted in many studies and has become a classical configuration in early PMD research and industrial applications. However, this model struggles to maintain accuracy when measuring complex specular surfaces with high curvature or sharp geometric variations, limiting its applicability in high-precision tasks.

To overcome the limited measurement flexibility inherent in the paraxial approximation model, researchers have proposed the reference-plane-based model, as illustrated in Figure 7b. This method assumes that the measured specular object is adjacent or approximately parallel to a known reference plane in space. By leveraging a geometric relationship among three key points—the image point P, the object point S, and the projection point Q—the surface gradient at point S can be derived. Compared to the paraxial approximation model, this model places fewer constraints on the geometric configuration of system components, offering greater flexibility. Huang et al. developed a fast measurement system based on this structure and used the Windowed Fourier Transform algorithm to achieve dynamic 3D reconstruction from a single-frame image, successfully capturing temporal deformations of water surface perturbations [96]. Li et al. further investigated the impact of reference plane positioning errors on measurement accuracy and introduced dual-laser-assisted positioning and confocal white-light distance sensors to improve spatial localization of the reference plane [97]. However, this model is mainly applicable to nearly flat surfaces. For objects with significant curvature or large deviations from the reference plane, its measurement accuracy degrades noticeably.

The surface estimation and reprojection model, as illustrated in Figure 7c, represents a more advanced modeling framework for PMD systems, specifically developed to address the challenges associated with measuring highly curved and complex surfaces.Unlike previous models, it does not rely on a flat reference plane or paraxial assumptions. Instead, it uses a coarsely estimated surface shape as a substitute for the reference plane and iteratively refines both surface shape and normal vectors based on reflective geometry principles. Within this framework, Bothe et al. achieved high-precision measurements for various highly reflective objects, including metals, transparent plastics, and glass, and demonstrated the model’s broad applicability to complex targets [98]. Su et al. developed the Software Configurable Optical Test System, which is used for 3D measurement of large curved mirrors in astronomical telescopes. The system iteratively improves measurement accuracy through reprojection optimization [99]. It is worth noting, however, that this model relies heavily on the accuracy of the initial surface estimate. Significant estimation errors can lead to substantial reconstruction deviations. To address this issue, some studies have used external devices such as coordinate measuring machines (CMM) to acquire coarse surface data. Nevertheless, achieving high-precision registration between the coordinate system of the CMM and the PMD system remains a critical challenge in practical deployment. In response, Xu et al. proposed a calibration method that integrates the manufacturing system and the PMD measurement system, directly establishing the spatial relationship between the two for real-time surface estimation in online measurement environments [100,101].

### 3.2. Multi-Screen Direct PMD

**Figure 8:**
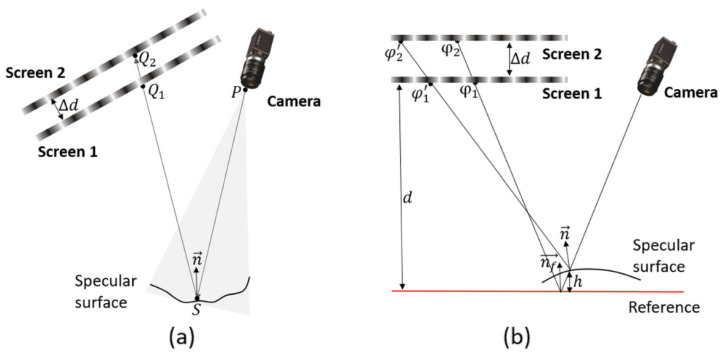
Schematic diagram of a multi-screen configuration in direct PMD systems. (**a**) Model based on screen movement. (**b**) Model based on DPMD [34].

To overcome the limitations of single-screen PMD systems in terms of surface normal estimation accuracy and visible measurement area, multi-screen configurations in direct PMD have been developed. As shown in Figure 8, such systems incorporate two or more display screens into the scene, allowing the viewing ray reflected from the measured point to pass through multiple known fringe patterns. This enables more stable and accurate reconstruction of surface normal [102,103,104].

A typical working principle of multi-screen PMD is illustrated in Figure 8a. Assume the camera’s viewing ray is reflected from a surface point *S* and sequentially passes through pixel positions $Q_{1}$ and $Q_{2}$ on two display screens. Given the known camera intrinsics and screen calibration data, the surface normal $\vec{n}$ at point *S* can be derived based on a ray reflection model. However, in practical implementations, the first screen may obstruct part of the optical path, preventing the camera from seeing the second screen directly. As a result, early systems often suffered from limited visibility and required specific geometric arrangements to overcome occlusion issues. Although this approach is effective, it significantly increases system complexity and measurement time, making it unsuitable for dynamic or real-time applications. To address this issue, Li et al. proposed an improved multi-screen PMD system based on a transparent display [105]. The core idea is to use a transparent screen as the front display, allowing the camera’s line of sight to pass through it and directly observe the fringe patterns on the second screen behind. This design enables simultaneous observation of two fixed screens without any mechanical movement, greatly simplifying the system structure, improving measurement efficiency, and enhancing adaptability for wide field-of-view measurements.

DPMD is an innovative specular surface 3D measurement technique proposed in recent years. Unlike traditional PMD, which relies on gradient field integration to reconstruct 3D shape, DPMD constructs symmetric reflection paths and directly obtains the phase difference in the surface under two different optical paths. This eliminates the need for complex integration and allows for direct computation of the object’s surface height [71,72]. In this method, the camera ray is sequentially reflected by a reference plane and the measured specular surface, intersecting fringe patterns displayed on two parallel screens. When fringe patterns are displayed on two parallel screens and reflected by a specular surface, four key phase values can be obtained: $\Phi_{1}$ and $\Phi_{2}$ along the reference path, and $\Phi_{1}^{\prime }$ and $\Phi_{2}^{\prime }$ along the object path. A schematic diagram (Figure 8b) is provided to illustrate the baseline distance *d* between the two parallel screens and the correction factor Δd accounting for possible system misalignment. Based on this geometry, the depth value *h* can be calculated as follows:

(23) $$h=\frac{d\left[\left(\Phi_{1}-\Phi_{2}\right)-\left(\Phi_{1}^{\prime }-\Phi_{2}^{\prime }\right)\right]-\Delta d\left(\Phi_{1}^{\prime }-\Phi_{1}\right)}{\left(\Phi_{2}-\Phi_{1}\right)+\left(\Phi_{2}^{\prime }-\Phi_{1}^{\prime }\right)}$$

In practical systems, to achieve symmetric phase acquisition, a parallel configuration is typically constructed using one physical screen and one virtual screen created by a beam splitter. However, ensuring strict parallelism between the virtual and physical screens remains challenging and may affect the overall system accuracy. Compared to traditional PMD, DPMD exhibits better adaptability and stability when measuring specular objects with large slope variations or discontinuities, making it particularly suitable for targets with step edges or abrupt surface changes. On the other hand, since DPMD does not rely on a complete gradient field, its measurement accuracy for smooth continuous surfaces is slightly lower than that of traditional PMD methods based on gradient integration.

### 3.3. Multi-Camera Stereo PMD

Stereo PMD is a specular surface 3D measurement technique based on multi-sensor collaborative imaging, first introduced by Knauer et al. in 2004 [93]. This method enables multiple cameras to observe the specular object synchronously from different viewpoints. By combining the phase information of the projected fringe patterns, surface normal is estimated from each viewpoint and then matched to reconstruct the 3D shape of the target object.

**Figure 9:**
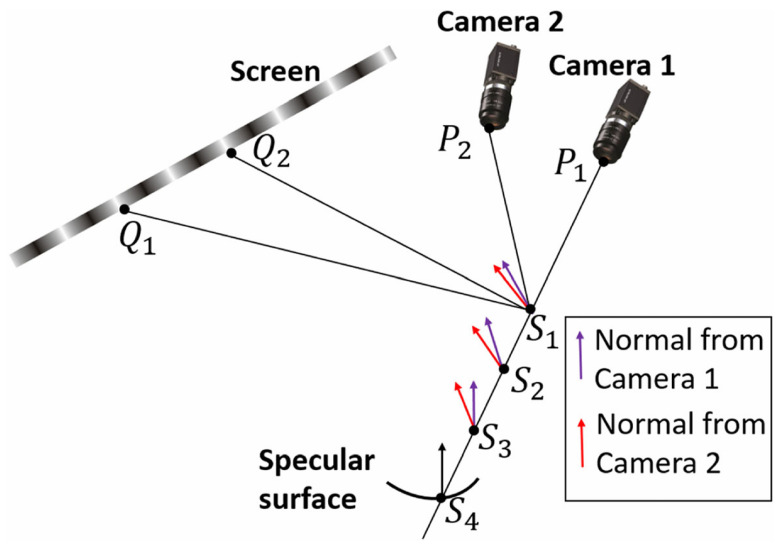
Illustration of stereo deflectometry [34].

As illustrated in Figure 9, a typical Stereo PMD system operates as follows: one primary camera selects a spatial point $S_{1}$, and, based on the system calibration parameters, determines its corresponding image point $P_{1}$ on the screen. The corresponding phase value at this location can then be retrieved from the screen’s phase map, allowing the reflected fringe point $Q_{1}$ to be identified. Using the three points $Q_{1}$, $S_{1}$, and $P_{1}$, the surface normal at point $S_{1}$ can be computed according to the law of reflection. Meanwhile, a secondary (auxiliary) camera also captures the same target point $S_{1}$, producing its own image point $P_{2}$. Following the same process, a second reflection point $Q_{2}$ is obtained, providing an independent estimation of the surface normal. Theoretically, the surface normals estimated from the two views should converge, allowing the recovery of the surface gradient through normal vector matching, and thereby enabling full 3D shape reconstruction. The main advantage of this method lies in its strong adaptability to surfaces with complex curvature, and its ability to achieve high reconstruction accuracy through normal matching. Studies have shown that Stereo PMD can achieve nanometer-level relative depth accuracy [106,107].

Furthermore, since it does not rely on a reference plane or prior surface estimation, it offers greater generalizability for applications involving large-scale specular surfaces or free-form reflective geometries.

## 4. Evolution and Advances of FPP Systems

**Figure 10:**
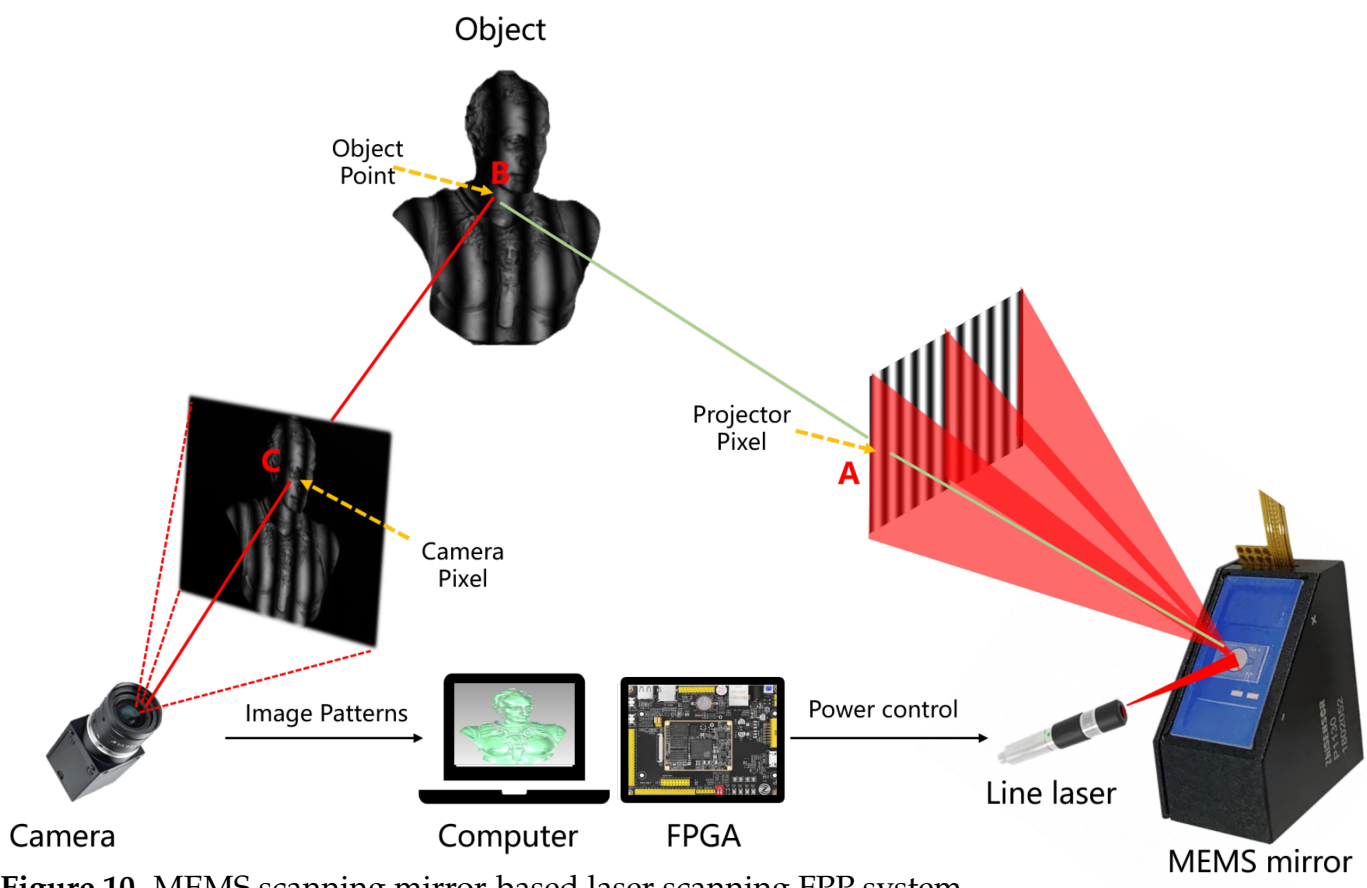
MEMS scanning mirror-based laser scanning FPP system.

In FPP systems, the projection module is the core front-end component for generating fringe patterns, and its performance directly determines key system metrics, including spatial resolution, projection speed, measurement depth of field, and environmental adaptability. With the continuous evolution of 3D measurement requirements—from static scenes to highly dynamic environments, from bulky setups to miniaturized devices, and from shallow-range measurements to large-depth tasks—traditional projection methods have exposed clear drawbacks. Specifically, focusing optics constrain the available depth of field, bulky hardware limits portability, and high sensitivity to ambient light reduces robustness in practical applications. In recent years, laser scanning projection technologies based on MEMS micromirrors as have attracted widespread attention due to their advantages in high precision, high speed, low power consumption, and compact structure. As shown in Figure 10, this technology achieves rapid deflection of laser beams and the generation of fringe patterns through MEMS micromirrors. Notably, the continuous advancement of MEMS technology has not only driven the development of novel projection architectures but also demonstrated strong compatibility with mainstream DLP-based systems. Given its high synergy with existing solutions and its tremendous potential in next-generation FPP systems, this chapter focuses on MEMS-based projection technologies and their applications in advanced structured-light systems.

### 4.1. Comparison of Mainstream Fringe Projection Technologies

**Figure 11:**
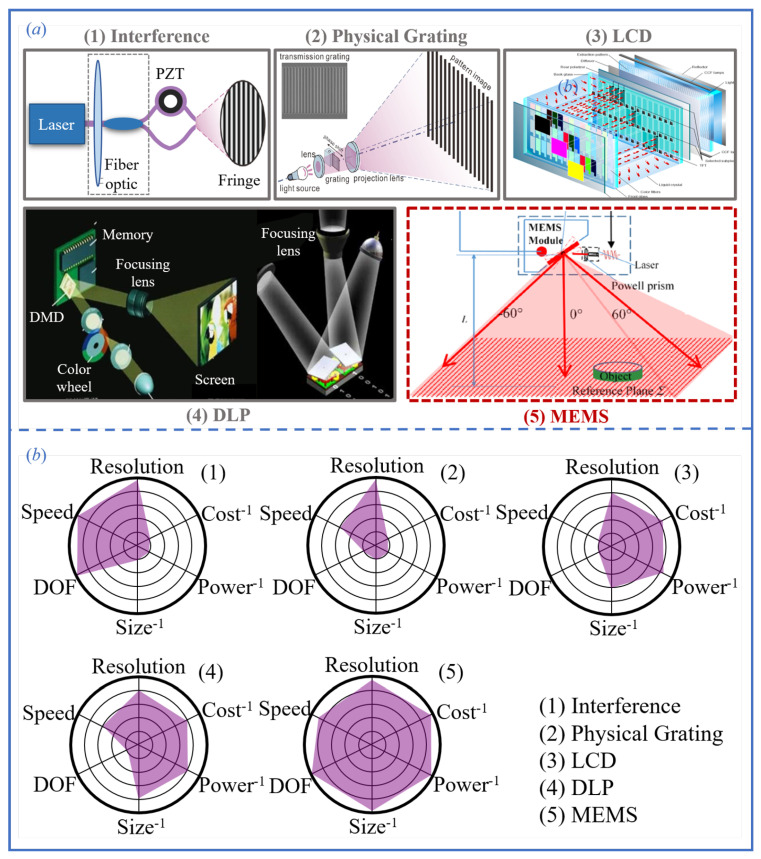
(**a**) Comparison of typical structured-light projection methods and their performance. (**b**) Radar charts comparing the performance of each projection method across multiple criteria [108].

In structured-light 3D measurement systems, the method of fringe pattern generation and the optical quality are among the most critical factors influencing reconstruction accuracy, robustness, and overall system performance. Different projection techniques exhibit significant differences in terms of fringe contrast, spatial resolution, refresh rate, and system size, all of which directly affect the stability of phase calculation and the system’s adaptability in dynamic or complex environments. As illustrated in Figure 11a, current mainstream fringe generation approaches can be roughly categorized into the following types: (1) optical interferometric projection, based on interference principles; (2) physical grating projection, using static optical gratings; (3) LCD-based pixel modulation projection, utilizing liquid crystal displays; (4) DLP digital projection, based on digital micromirror devices (DMD); (5) MEMS micromirror-based laser scanning projection, using micro-electro-mechanical systems. Each method has its own characteristics in terms of pattern flexibility, system complexity, cost, and suitable application scenarios. Among them, DLP projection has become the most widely adopted technique due to its high pattern flexibility and strong grayscale modulation capability. However, it typically relies on projection lenses for focusing, which limits the depth of field of the system. In contrast, MEMS micromirror projection generates fringe patterns by directly scanning a laser beam in space. This approach requires no focusing optics, and offers distinct advantages such as large depth of field, compact size, low power consumption, and mechanical simplicity, making it particularly well-suited for embedded systems, dynamic scenes, and mobile platform-based 3D measurement applications.

To further compare and analyze the practical performance of the five aforementioned structured-light projection methods, Figure 11b presents a radar chart evaluating their capabilities across five key dimensions: resolution, speed, depth of field, system size, and cost. Additionally, Table 2 summarizes the representative technical specifications of each system.

From the chart and table, it can be observed that interference-based structured-light systems generate fringe patterns through coherent beam interference, achieving sub-micron spatial resolution and excellent depth-of-field performance. These characteristics make them particularly suitable for measurements of micro/nano-scale structures and the characterization of curved surface topographies. However, such systems lack pattern programmability, impose strict requirements on environmental stability, involve complex system construction, and incur high costs, all of which limit their practical applicability. Structured-light systems based on physical gratings generate periodic fringe patterns by combining fixed grid structures with illumination sources. These systems are characterized by simple configuration and stable fringe quality, making them suitable for static measurement scenarios requiring high accuracy [109]. However, their fringe patterns are not programmable, which limits their ability to implement multi-frequency modulation or adaptive pattern adjustments. Consequently, their flexibility is significantly constrained. Furthermore, similar to interference-based projection methods, physical grating systems are non-digital and are thus inadequate for applications requiring a high diversity of fringe encodings or precise modulation in dynamic and complex environments. LCD-based structured-light systems modulate patterns by controlling the transmittance of liquid crystal elements. These systems offer advantages such as low cost, design flexibility, and low power consumption, making them well-suited for mass production at scale [110]. Nevertheless, the slow response speed of liquid crystal elements and their limited grayscale control capability result in insufficient sharpness and refresh rates for high-speed and high-precision measurements.

In addition, the pixelated structure of LCD panels introduces non-ideal responses under high-frequency fringe patterns, which negatively affects phase demodulation accuracy, thereby limiting their applicability in industrial precision inspection. DLP projection systems are currently the most mainstream digital implementation of structured-light technology. Their core component is the digital micromirror device (DMD), which enables high refresh rates, support for arbitrary pattern projection, and multi-level grayscale control [111]. In standard 8-bit mode, DLP projectors can achieve projection rates on the order of hundreds of frames per second while maintaining excellent pattern consistency and spatial resolution, making them suitable for most static and low-speed dynamic 3D reconstruction tasks. To overcome speed limitations, some studies have proposed the use of one-bit binary defocused projection strategies, enabling projection rates of over one thousand frames per second while maintaining acceptable pattern fidelity. However, DLP systems are typically equipped with front-end focusing lenses, which limit their depth of field and make them unsuitable for targets with significant depth variation or pronounced surface curvature. Moreover, the complex optical layout, large physical footprint, and high cost of DMD components present challenges for integration into portable or embedded systems. MEMS-based micromirror projection technology has steadily matured in recent years. By using single- or dual-axis resonant micromirrors to scan laser beams and generate two-dimensional fringe patterns, MEMS systems offer a significant advantage in that they can form sharp patterns without the need for focusing optics. This enables designs with ultra-large depth of field, compact size, and low power consumption. Additionally, MEMS projectors can dynamically control laser power via high-speed TTL or analog modulation, supporting wide dynamic range and frequency-controllable pattern generation. These features enable excellent real-time performance and high frame rates, making MEMS systems particularly well-suited for mobile platforms, robotic grasping, and dynamic 3D perception tasks. Owing to their beam controllability and miniaturized structure, MEMS-based solutions provide essential hardware support for the development of lightweight and intelligent structured-light systems.

In summary, MEMS-based micromirror projection technology demonstrates exceptional system integrability and environmental adaptability, owing to its lens-free configuration, large depth of field, compact size, low power consumption, and high-speed performance. These characteristics make it particularly well-suited for space-constrained, mobile, or dynamic 3D reconstruction scenarios. By employing laser beam scanning to directly render fringe patterns, MEMS projectors achieve a seamless integration of pattern precision and flexibility, effectively overcoming the trade-off constraints among volume, depth of field, and resolution typically encountered in traditional lens-based projection systems. With ongoing advancements in MEMS device fabrication precision and control algorithms, MEMS-based structured-light projection is emerging as a strong contender to DLP technology, driving the evolution of 3D reconstruction systems toward higher precision, greater miniaturization, and enhanced intelligence.

### 4.2. System Calibration Strategies for MEMS-Based Structured-Light Systems

Conventional structured-light systems typically employ the Phase-Height Model and the Triangulation Model for system calibration, as thoroughly reviewed in Section 2.3.2 [74]. However, due to the fundamental differences in physical mechanisms, calibration models must be adapted accordingly [112]. In particular, MEMS-scanned structured-light systems differ significantly in their projection principles and fringe generation mechanisms, rendering traditional pinhole-based projector models unsuitable for direct application. Specifically, in MEMS systems, fringe patterns are generated by a laser beam rapidly scanned by micromirrors, resulting in a dynamically varying beam incidence direction rather than a fixed projection center as in conventional projectors. This dynamic “point–line–plane” projection mechanism necessitates calibration models that incorporate the nonlinear relationship among laser scanning angles, power modulation, and camera imaging.

The design of calibration models for MEMS-based structured-light systems must take into account two essential characteristics of their projection modules: (1) the absence of focusing lenses and (2) unidirectional scanning projection [113,114]. To address these challenges, several studies have proposed calibration models tailored to MEMS micromirror scanning mechanisms. The following section introduces three representative modeling approaches: the unified model, the iso-phase surface model, and the phase-angle model.

#### 4.2.1. Joint Calibration Model

**Figure 12:**
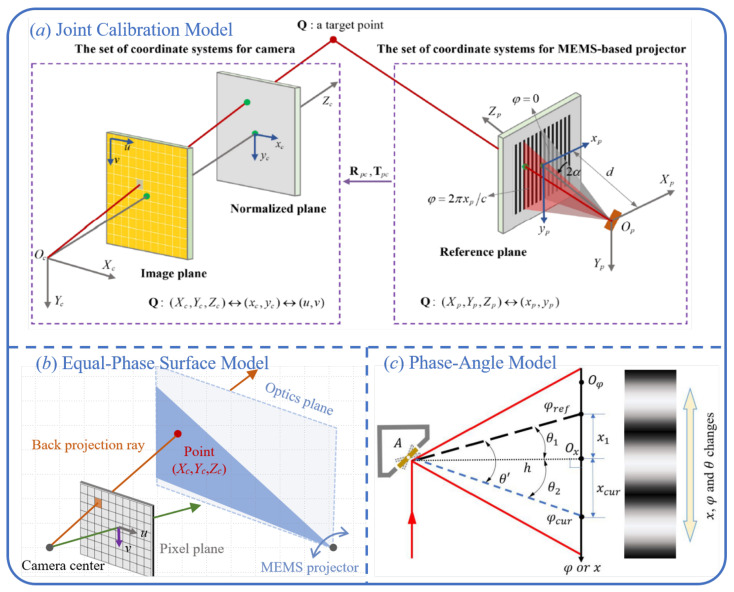
Three calibration models of MEMS-based structured-light systems. (**a**) Joint calibration model. (**b**) Equal-phase surface model. (**c**) Phase-angle model [108].

To address the projection characteristics of MEMS micromirror-based structured-light systems, the unified model provides a physically grounded and high-precision calibration strategy. As illustrated in Figure 12a, the core idea of the unified model is to couple the spatial coordinates of the MEMS laser scanning system with the camera imaging model under a common coordinate framework, thereby establishing an analytical mapping from phase values to 3D point coordinates [32].

Since the MEMS projection process essentially involves laser beam scanning along a defined plane, it can be assumed that the spatial positions of the projected points lie on a scanning plane subject to linear constraints. Given the known distance *d* between the reference plane and the initial projection point, and incorporating the geometric constraints of the laser scanning trajectory, the position of the projected point in the projector coordinate system can be derived as follows:

(24) $$\left[\begin{array}{c} x_{p}\\ y_{p} \end{array}\right]=\frac{d}{Z_{p}}\left[\begin{array}{c} X_{p}\\ Y_{p} \end{array}\right]$$

Meanwhile, the camera imaging process can be described by the standard pinhole projection model [82]:

(25) $$\left[\begin{array}{c} x_{c}\\ y_{c} \end{array}\right]=\frac{1}{Z_{c}}\left[\begin{array}{c} X_{c}\\ Y_{c} \end{array}\right]$$

By applying the rotation matrix $R_{pc}$ and translation vector $T_{pc}$ to align the coordinate systems of the projector and the camera, a unified representation in the world coordinate system can be obtained:

(26) $$\left[\begin{array}{c} X_{c}\\ Y_{c}\\ Z_{c} \end{array}\right]=R_{pc}\left[\begin{array}{c} X_{p}\\ Y_{p}\\ Z_{p} \end{array}\right]+T_{pc}$$

Combining Equations (23)–(25) and eliminating the intermediate variables $y_{p}$, $X_{p}$, $Y_{p}$, $Z_{p}$, $X_{c}$, $Y_{c}$, one can derive the following expression:

(27) $$Z_{c}=\frac{\begin{array}{c} \left(r_{22}r_{33}-r_{23}r_{32}\right)t_{1}+\left(r_{13}r_{32}-r_{12}r_{33}\right)t_{2}+\left(r_{12}r_{23}-r_{13}r_{22}\right)t_{3}+\\ (r_{12}r_{23}-r_{13}r_{22}+\left(r_{22}r_{33}-r_{23}r_{33}\right)x_{c}+\left(r_{12}r_{21}-r_{11}r_{22}\right)x_{p}/d \end{array}}{\begin{array}{c} \left(\left(r_{22}r_{31}-r_{21}r_{32}\right)t_{1}+\left(r_{11}r_{32}-r_{12}r_{31}\right)t_{2}+\left(r_{12}r_{21}-r_{11}r_{22}\right)t_{3}\right)x_{p}/d+\\ \left(r_{22}r_{31}-r_{21}r_{32}\right)x_{c}x_{p}/d+\left(r_{13}r_{32}-r_{12}r_{33}\right)y_{c}+\left(r_{11}r_{32}-r_{12}r_{31}\right)y_{c}x_{p}/d \end{array}}$$

where $r_{ij}$ and $t_{i}$ represent the elements of the rotation matrix and translation vector, respectively. Each scanning position $x_{p}$ corresponds to a phase value φ, with $\varphi =2\pi x_{p}/c$, where *c* denotes the preset fringe period constant. By substituting this phase relationship into the geometric expression of the unified model and consolidating the constant terms, two interpretable calibration models for MEMS-based structured-light systems can be further derived.

The first type is the global calibration model, which assumes that all pixels in the system share the same set of geometric and system parameters. In this model, all constant terms are incorporated into a single expression, providing a concise formulation that describes the pixel depth $Z_{c}$ as a function of image coordinates $\left(x_{c},y_{c}\right)$ and phase value φ

(28) $$Z_{c}=\frac{a_{1}+a_{2}\Phi }{a_{3}+a_{4}x_{c}+a_{5}\Phi +a_{6}x_{c}\Phi +a_{7}y_{c}+a_{8}y_{c}\Phi }$$

The second type is the per-pixel calibration model, which relaxes the unified parameter constraints imposed by the global model. This approach assumes that each pixel possesses an independent set of calibration parameters. Accordingly, in practical modeling, the constants associated with each pixel can be extracted and combined with other terms to form the following per-pixel expression [32].

(29) $$Z_{c}=\frac{a_{1}^{\prime }+a_{2}^{\prime }\Phi }{a_{3}^{\prime }+a_{4}^{\prime }\Phi }$$

In both models, the unknown calibration parameters are typically solved using a linear least-squares method in conjunction with a system of homogeneous equations [74,78]. Since the above derivations are based on ideal image coordinates, while real imaging processes inevitably introduce camera distortion, it is necessary to first use the camera calibration results to convert the distorted image coordinates into ideal ones in order to ensure modeling accuracy. Moreover, MEMS-based structured-light systems generally adopt a lensless projection design, which eliminates projection distortions caused by optical lenses in the projection path, thereby simplifying the geometric modeling process.

#### 4.2.2. Equal-Phase Surface Model

To address the calibration challenges arising from the unique imaging structure of MEMS-based projection systems, Miao et al. proposed the isophase plane model [115]. This method constructs a series of isophase light planes formed by laser scanning and leverages the geometric relationship between the camera’s imaging center and the surface reflection point to achieve pixel-wise 3D coordinate estimation. As illustrated in Figure 12b, the isophase planes can be regarded as a set of approximately parallel light planes generated at specific scanning angles. Each plane is associated with a unique phase value. By correlating the phase information received at a particular camera pixel, the intersection point between the reflected light ray and the corresponding isophase plane can be determined. Subsequently, the 3D coordinate of the measured point is derived by fitting the reflection path between the camera and the isophase planes, resulting in a mapping function between the phase and spatial coordinates.

(30) $$X_{c}=\frac{1}{\sum_{n=0}^{N}a_{n}\Phi^{n}}+a_{X},\;Y_{c}=\frac{1}{\sum_{n=0}^{N}b_{n}\Phi^{n}}+b_{Y},\;Z_{c}=\frac{1}{\sum_{n=0}^{N}c_{n}\Phi^{n}}+c_{Z}$$

This method fully accounts for image distortion effects in the calibration modeling process; as a result, higher-order polynomials are often introduced in the denominator of the mapping expressions. However, to prevent overfitting caused by excessive model complexity, it is essential to carefully select the polynomial order [74]. Currently, two primary approaches are used to mitigate the impact of image distortion on system calibration. The first is based on the actual image coordinates and employs polynomial fitting to suppress image noise. The second approach involves converting all image coordinates into ideal coordinates using the intrinsic camera parameters before performing modeling. Experimental results have shown that both strategies can achieve satisfactory calibration performance in MEMS-based structured-light systems.

#### 4.2.3. Phase-Angle Model

As shown in Figure 12c, the phase-angle model is a calibration method that directly relates the geometry of laser beam propagation to phase information, and it is particularly well-suited for laser scanning projection mechanisms commonly found in MEMS-based structured-light systems [116,117]. During the scanning process of a MEMS micromirror, each isophase position corresponds to a unique scanning angle. Therefore, given a known phase value and combined with geometric constraints, the three-dimensional spatial coordinates of a specific reflection point can be inferred.

In this model, the laser beam at a specific phase value φ corresponds to a unique scanning direction, and the reflected rays associated with different phase values exhibit a strictly linear relationship along the projection path. Based on this, and in combination with the camera imaging model, a direct mapping can be established between the pixel coordinates (u,v), the phase Φ, and the spatial coordinates $\left(X_{c},Y_{c},Z_{c}\right)$ of the reflection point on the measured object. By introducing auxiliary parameters *A*, *B*, *C*, and *D*, the complex geometric computation can be simplified into the following expression:

(31) $$X_{c}=\frac{-uD}{uA+vB+C},\;Y_{c}=\frac{-vD}{uA+vB+C},\;Z_{c}=\frac{-D}{uA+vB+C}$$

The primary advantage of this method lies in its independence from the need for an explicit calibration board that fully covers the camera’s field of view, as well as from the complex gradient integration process required in traditional models. This significantly simplifies the system calibration workflow. Additionally, the phase-angle model demands relatively low quantities and precision of image acquisition, allowing for complete system calibration using only a subset of captured images. This makes it particularly suitable for embedded systems or online measurement scenarios where computational resources and time are constrained.

### 4.3. Analysis of Systematic and Random Errors

Similar to conventional structured-light systems, MEMS-based structured-light systems are also subject to a range of common error sources. However, due to their distinctive laser scanning principles and physical implementation mechanisms, MEMS systems exhibit a series of unique error factors. These errors manifest throughout various stages of the 3D reconstruction pipeline, spanning from fringe pattern projection and image acquisition to phase extraction and the final generation of 3D point clouds [118,119,120,121,122,123]. Specifically, the error sources in MEMS structured-light systems include unstable motion of the scanning mirror, non-ideal line width of the laser stripe [124], noise from the laser source, initial phase shift errors in the mechanical rotation of the scanning mirror, and misalignment between the laser optical axis and the scanning mirror’s rotational axis [116].

#### 4.3.1. Random Errors

**Figure 13:**
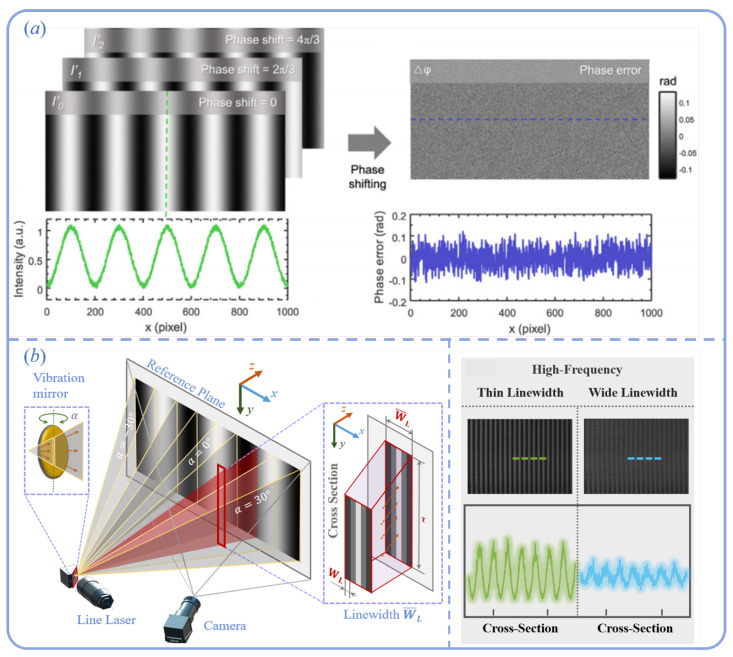
Illustration of two factors affecting phase accuracy in structured-light systems. (**a**) Random intensity noise. (**b**) Non-ideal line width smoothing effect [108].

In the process of structured-light 3D reconstruction, fringe pattern projection and image acquisition are two core components. Random noise, as an inevitable source of disturbance, can significantly impact the accuracy of phase extraction and 3D reconstruction. In MEMS-based structured-light systems, the primary sources of random errors include intensity fluctuations of the laser (source noise), imaging noise during camera acquisition (such as readout noise and dark current noise), and temporal or spatial jitter induced by instability during the resonant scanning of the MEMS micromirror. These noise sources manifest in the captured images as localized or global grayscale disturbances, leading to random deviations in the computed phase. In phase calculation, when using the N-step phase-shifting method to extract wrapped phase, random noise directly affects the brightness distribution of each captured frame, as illustrated in Figure 13a.

The corresponding modulation model can be described as follows [125]:

(32) $$I_{n}=A+B\cos\left(\varphi -\delta_{n}\right)=A_{0}+\Delta I+B\cos\left(\varphi -\delta_{n}\right)$$

where $A_{0}$ denotes the background intensity, *B* represents the modulation depth, φ is the ideal phase, $\delta_{n}$ is the phase shift, and ΔI refers to additive Gaussian white noise with zero mean and standard deviation $\sigma_{n}$. Based on least-squares derivation, it can be shown that the noise introduces phase errors, with a standard deviation given by the following [125,126]:

(33) $$\sigma_{\varphi }=\sqrt{\frac{2}{N}\cdot \frac{\sigma_{n}}{B}}$$

If the fringe frequency is *f*, then after phase unwrapping, the phase range expands from 2π to 2πf. When the absolute phase is compressed back to the range of [−π,π), the standard deviation of the phase error becomes the following:

(34) $$\sigma_{\varphi }=\sqrt{\frac{2}{N}\cdot \frac{\sigma_{n}}{Bf}}$$

To effectively mitigate the impact of random noise on the 3D reconstruction accuracy of MEMS-based structured-light systems, three optimization strategies can be considered. First, increasing the number of phase shifts can significantly enhance the robustness of phase computation and reduce noise-induced fluctuations; however, this also leads to longer acquisition times, which may compromise system efficiency. Second, improving the modulation depth of the fringe pattern is another effective means of reducing phase errors. It is important to note that the line laser used in MEMS systems is not an ideal infinitesimal beam but possesses a finite width—referred to as the “non-ideal line width”—which differs from the fringe period. This non-ideal width causes a “window smoothing effect” on the fringe pattern, thereby reducing the modulation depth. As illustrated in Figure 13b, this effect significantly degrades fringe contrast and phase quality. To address this issue, Han et al. proposed a window smoothing model and developed an optimal fringe number recommendation algorithm that can automatically determine the most suitable fringe frequency combination based on system parameters to achieve optimal reconstruction performance [124]. Finally, reducing image-level random noise is also crucial for improving phase stability. In recent years, with advances in deep learning, convolutional neural network (CNN)-based image denoising techniques [127] have been widely applied in structured-light systems. These methods can effectively suppress random noise while preserving image details, thereby further enhancing the accuracy and robustness of 3D reconstruction.

#### 4.3.2. Impact of Line Laser Intensity Fluctuations

The standard phase-shifting method typically assumes that the background intensity and modulation amplitude remain constant across different phase-shifted fringe patterns at the same pixel location. However, in practical measurement environments, this assumption often does not hold due to fluctuations in the intensity of the line laser source. Such intensity fluctuations cause variations in both the background illumination and the modulation amplitude across phase-shifted images, which in turn lead to phase errors. The influence of light intensity fluctuations on phase-shifted fringes can be modeled using the following equation:

(35) $$I_{n}=p_{n}\left[A+B\cos(\phi -\delta_{n})\right]+q_{n}$$

where $p_{n}$ denotes the proportional coefficient of the line laser intensity fluctuation, and $q_{n}$ represents the additive component of the fluctuation. These two factors cause variations in the background intensity or modulation amplitude across different phase-shifted fringe images, thereby introducing phase errors. By substituting the phase-shifted images—with both background intensity offsets and modulation amplitude deviations—into the *N*-step phase-shifting expression, the resulting phase error can be derived [128]:

(36) $$\Delta \phi \approx \frac{2}{NB}\sum_{n=1}^{N}\frac{\left(p_{n}-1\right)A+q_{n}}{p_{n}}\sin\left(\delta_{n}-\phi \right)$$

To mitigate the impact of line laser intensity fluctuations on 3D reconstruction accuracy, the most direct hardware-level solution is to employ a laser source with stable output. A stable laser can fundamentally reduce intensity variations at the source, thereby avoiding phase extraction errors caused by light source instability. On the software level, post-processing techniques can effectively compensate for errors induced by intensity fluctuations. For instance, Liu et al. proposed an iterative self-calibration algorithm that rapidly extracts the phase components from fringe images and accurately compensates for deviations in background intensity and modulation amplitude [129]. This method enhances phase extraction accuracy through iterative optimization and maintains robust reconstruction performance even under unstable illumination. In addition, Lu et al. developed a histogram-based segmentation approach, in which each phase-shifted image is segmented and corrected via a linear gray-level transformation to compensate for background intensity and modulation amplitude shifts [130]. By adjusting the gray levels, this method effectively eliminates deviations caused by intensity fluctuations, thereby improving phase accuracy. Chen et al. proposed two real-time correction methods specifically designed to address source instability [131]. These techniques utilize dynamic mapping functions to correct phase errors in real time as they evolve over time. Such correction strategies not only counteract the influence of an unstable light source but also enable adaptive adjustment in dynamic environments, ultimately enhancing the precision of 3D reconstruction.

#### 4.3.3. High-Order Harmonics

In traditional structured-light systems, the Gamma effect or system nonlinearities typically introduce higher-order harmonic errors [132,133]. This issue becomes even more pronounced in emerging structured-light systems based on MEMS micromirror scanners, where mechanical rotational errors of the mirror lead to the coupling of higher-order harmonics into the projected fringe patterns. Furthermore, when the input–output characteristics of the laser source are not accurately calibrated, similar harmonic distortions may arise. The presence of higher-order harmonics contaminates the captured images, distorting the fringe patterns and thus compromising the accuracy of phase extraction. These distortions can be mathematically described using a Fourier series expansion [118]. Let $a_{i}$ denote the amplitude of the *i*-th harmonic component; then, the distorted fringe image containing higher-order harmonics can be expressed as follows:

(37) $$I_{n}^{c}\left(x,y\right)=a_{0}+\sum_{m=1}^{\infty }a_{i}\cos\left(i\left(\phi (x,y)\right)+\varphi_{n}\right)$$

When using the N-step phase-shifting method for phase extraction, the phase error introduced by higher-order harmonics can be derived using the following expression [119,120,121]:

(38) $$\Delta \phi =\tan^{-1}\left[\frac{\sum_{m=1}^{\infty }\left(a_{mN+1}-a_{mN-1}\right)\sin\left(mN\phi (x,y)\right)}{a_{1}+\sum_{m=1}^{\infty }\left(a_{mN+1}-a_{mN-1}\right)\cos\left(mN\phi (x,y)\right)}\right]$$

To mitigate the impact of higher-order harmonics on phase accuracy, one commonly adopted strategy is to increase the number of phase-shifting steps, which can effectively suppress harmonic interference. However, this inevitably leads to an increased number of required images, thereby reducing the overall reconstruction speed [122]. Therefore, the most meaningful approach is to suppress higher-order harmonic effects without significantly compromising the reconstruction efficiency.

Harmonic suppression strategies can generally be categorized into two types: active methods and passive methods. Active methods involve pre-calibration before pattern projection, whereas passive methods are implemented after the projection has occurred [123]. Specifically, Huang et al. proposed a dual three-step phase-shifting technique that enhances phase measurement accuracy by optimizing the conventional three-step phase-shifting method [134]. Cai et al. derived phase error models in both the spatial domain and the Hough Transform (HT) domain, which are used to analyze and compensate for the effects of higher-order harmonics on phase extraction [118]. Zhang et al. employed a lookup-table-based approach to correct the nonlinear errors in projectors [135]. Furthermore, Pan et al. conducted theoretical analysis on phase errors caused by non-sinusoidal waveforms and developed an iterative phase compensation algorithm to effectively reduce the impact of higher-order harmonics [136]. Song et al. proposed a system nonlinearity correction method based on mask information, where harmonic coefficients are determined using a mask image and the true phase is recovered through Gauss–Newton iteration [137].

While these methods have been extensively applied in conventional DLP-based structured-light systems, harmonic suppression techniques specifically designed for MEMS-based systems remain relatively scarce. To address this, Han et al. proposed a layered phase-shifting method based on a phase-shifting superposition framework, leveraging the fact that MEMS scanning speed is typically higher than that of the camera [138]. While these methods have been extensively applied in conventional DLP-based structured-light systems, harmonic suppression techniques specifically designed for MEMS-based systems remain relatively scarce. To address this, Han et al. proposed a layered phase-shifting method based on a phase-shifting superposition framework, leveraging the fact that MEMS scanning speed is typically higher than that of the camera.

**Figure 14:**
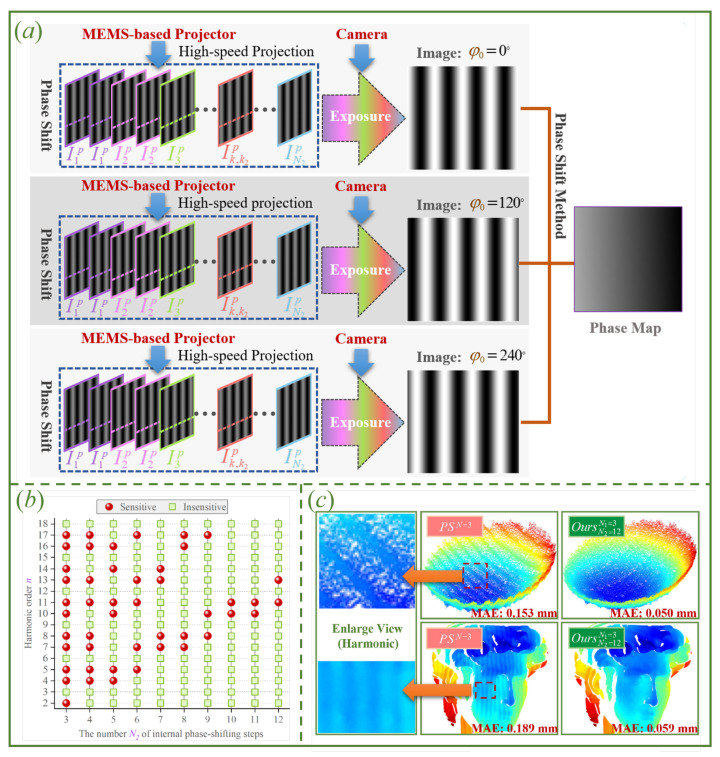
Layered phase-shifting method proposed by Han et al. (**a**) Principle of the layered phase-shifting method. (**b**) Sensitivity of the internal phase-shifting method to harmonic distortions. (**c**) Experimental results of the nested (internal–external) phase-shifting method [108].

As illustrated in Figure 14a, the internal phase-shifting method projects 12 phase-shifted patterns within a single camera exposure period. These patterns are temporally superimposed into a single image using the camera’s exposure integration, effectively suppressing harmonic distortions. The external phase-shifting method then extracts the wrapped phase from these harmonic-free composite images. Experimental results demonstrate that this approach achieves the same accuracy as a conventional 12-step phase-shifting method, while requiring only three captured images. Figure 14b illustrates the sensitivity of various harmonic orders to different internal phase-shifting step counts. Figure 14c compares the 3D reconstruction results obtained by the traditional three-step phase-shifting method and the proposed layered phase-shifting method with 3 external and 12 internal steps.

Despite the continuous progress of traditional FPP methodologies—including innovations in phase extraction, unwrapping, and calibration—these approaches remain constrained by hardware limitations, sensitivity to ambient noise, and reduced robustness in low-contrast or large-depth-of-field scenarios. At present, deep learning techniques have already been validated and applied in many domains [139,140,141]. They are capable of complementing or even surpassing traditional models by automatically learning robust representations from large-scale datasets. In this context, deep learning has emerged not only as a tool for improving accuracy and efficiency [142,143,144,145], but also as a transformative paradigm for addressing longstanding issues in fringe-structured-light reconstruction. The following section provides a systematic overview of how deep learning frameworks have been designed and adapted to meet these challenges.

## 5. Application of Deep Learning in Fringe-Structured Light

In traditional fringe-structured-light systems, measurement accuracy often faces significant challenges when dealing with objects that exhibit non-uniform surface reflectivity [146], complex geometries, or severe occlusions [147]. In recent years, deep learning techniques have been extensively validated and successfully applied across various fields, demonstrating powerful capabilities in feature extraction and nonlinear modeling [44,140,148,149,150]. Specifically, for fringe-based structured-light systems, deep learning offers novel solutions to improve measurement accuracy, accelerate reconstruction speed, and enhance system robustness. This chapter provides a detailed overview of the applications of deep learning in fringe projection-based structured-light systems. However, since PMD primarily targets specular objects and is constrained by its specific application scenarios, the use of deep learning in PMD remains limited. Existing studies are usually centered on single-shot approaches [151,152,153,154,155,156,157,158]. Therefore, this chapter mainly focuses on deep learning-driven FPP methods.

### 5.1. Learning Paradigm for Deep Learning-Driven FPP

**Figure 15:**
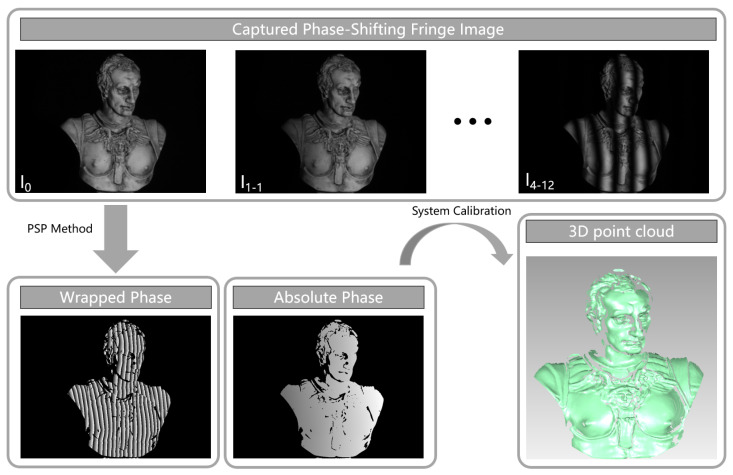
Schematic diagram of the traditional phase-shifting measurement process.

Deep learning-based approaches can be categorized into two types—single-frame methods and multi-frame methods—following the classification of traditional phase-shifting [45,46] and Fourier-based algorithms [49,159]. In traditional multi-frame phase-shifting techniques, multiple fringe images are acquired to enhance the accuracy and robustness of phase recovery by leveraging temporal redundancy, As illustrated in Figure 15. Here, $I_{0}$ denotes the original scanned object, $I_{1-1}$ denotes the first step of the first frequency, and $I_{4-12}$ denotes the 12th step of the fourth frequency. These methods have been thoroughly discussed in Section 2.1.

In the context of multi-frame fringe projection 3D reconstruction, deep learning models aim to learn the mapping between multi-frequency fringe patterns and depth information. Unlike conventional methods that explicitly extract phase information from the fringe images [45,46], deep learning approaches use neural networks to automatically establish this mapping, thus reducing the need for handcrafted feature design and enabling more efficient and accurate phase recovery. Such models are particularly beneficial in handling complex measurement scenarios, as they reduce acquisition redundancy while improving reconstruction speed and precision.

In contrast, single-frame approaches are inherently more challenging, as the deep learning model must infer phase information from only one fringe image. Traditional single-frame phase retrieval techniques rely on frequency domain analysis to extract phase [49,159]. However, deep learning-based single-frame models do not depend on explicit geometric constraints or analytical markers; instead, they utilize implicit features learned from large-scale datasets to recover phase information. By capturing local phase distributions and the inherent structure of the fringe image, these models can robustly estimate phase even under challenging lighting conditions, such as shadows and occlusions.

In summary, the integration of deep learning into fringe projection-based 3D reconstruction has led to significant performance improvements for both single-frame and multi-frame scenarios. Whether enhancing traditional multi-frame phase-shifting methods or addressing the complexities of single-frame phase recovery, deep learning models enable more efficient, robust, and accurate solutions for phase retrieval and depth estimation.

### 5.2. Deep Learning Framework Design and Advancements

Current fringe-to-phase/depth methods are primarily distinguished by three technical dimensions: network architecture, supervision strategy, and input paradigm.

#### 5.2.1. Network Architecture Innovations

In deep learning-driven fringe-structured-light 3D reconstruction, designing an effective framework to map fringe images into phase information is of paramount importance. Current research has primarily focused on innovations in neural network architectures, particularly models tailored for fringe-to-phase regression tasks. Since this mapping is essentially a regression problem, U-Net and its variants have become the dominant approaches. By leveraging skip connections for hierarchical feature integration, U-Net effectively captures both local and global context. Comparative studies [160,161] have demonstrated that U-Net achieves higher prediction accuracy and stability than traditional CNNs and GANs. However, these benefits often come at the cost of increased computational complexity and limited cross-domain adaptability, which has motivated further architectural refinements.

Recent advances have extended the U-Net backbone with new design concepts, hybrid strategies, and pretrained modules to improve accuracy, reduce training time, and enhance generalization. For example, Wang et al. [162] proposed MSUNet++, which incorporates additional nested pathways to fuse features across multiple levels, thereby enhancing representational power for complex mappings. This improvement, however, comes with longer training and inference times. Zhu et al. [163] developed PCTNet, a CNN–Transformer hybrid network that combines local texture extraction with global context modeling. Recognized as a state-of-the-art (SOTA) method in 2023, PCTNet achieved a 43.62% reduction in RMSE compared with U-Net, highlighting the advantages of hybrid architectures and further advancing research in the field.

Another promising direction is the integration of pretrained models. Li et al. [164] and Cai [165] introduced pretrained ResNet and Vision Transformer initializations into U-Net variants, both of which outperformed the conventional U-Net. Notably, Cai et al. employed pretrained Vision Transformers to extract semantically rich contour features and coarse depth cues, reducing MAE by 65%. These results indicate that pretrained models not only accelerate convergence but also substantially improve data efficiency, which is especially valuable in FPP systems where dataset sizes are typically limited.

Overall, existing evidence suggests that hybrid architectures combining the local feature extraction capabilities of CNNs with the global context modeling strength of Transformers deliver the most balanced performance. Although such models generally incur higher computational costs, they are particularly effective in handling complex scenarios where robustness and data efficiency are crucial. Consequently, pretrained visual models have emerged as an important tool for improving the performance of fringe-structured-light 3D reconstruction under data-constrained conditions.

Architectural innovations have thus laid a solid foundation for FPP and delivered significant improvements in accuracy and robustness. Nevertheless, relying solely on architectural advances remains insufficient to fully overcome the bottlenecks caused by limited samples and restricted input information. In recent years, researchers have begun to explore more sophisticated supervision strategies, introducing multi-level supervisory signals or incorporating physical priors during training to further enhance generalization and stability. The next section will focus on the latest developments in these supervision mechanisms.

#### 5.2.2. Supervision Strategies

**Figure 16:**
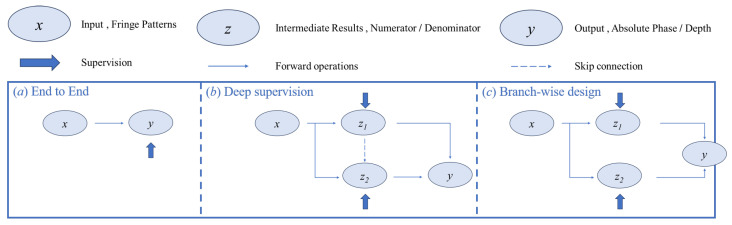
Different network supervision mechanisms. (**a**) End-to-End supervision. (**b**) Deep supervision. (**c**) Branch-wise design.

Although architectural innovations have improved baseline performance, conventional end-to-end learning still faces difficulties when addressing the inherent challenges of FPP, such as limited input information and small-scale datasets. As shown in Figure 16b,c, recent studies have increasingly incorporated physical priors in combination with tailored supervision strategies, which have proven to be effective in overcoming these bottlenecks and enhancing model performance.

Representative of these efforts is deep supervision, which introduces supervisory signals at multiple levels. This approach not only alleviates shortcut learning but also regularizes hierarchical feature representations, thereby significantly improving model generalization and enabling more robust reconstruction in complex scenarios [166,167,168]. For instance, Nguyen et al. [169] implemented multi-level supervision in the hNet architecture by injecting supervisory signals at each decoding stage, which markedly improved feature learning and consistently outperformed the conventional U-Net across most applications. Inspired by MFTPU, Li et al. [164] proposed the DSAS architecture, which applies joint supervision between sub-high-frequency absolute phase and high-frequency wrapped phase. Compared with standard end-to-end methods, DSAS reduced mean absolute error (MAE) in absolute phase reconstruction by 34%. Extending this idea, Zhu et al. [170] proposed a triple-supervision mechanism, which added an additional supervisory branch beyond the dual-branch design. This method lowered mean squared error (MSE) by 52% relative to end-to-end learning, further pushing the performance boundary.

Another line of research is branch-wise supervision, which combines network predictions with physical equations. Unlike deep supervision, branch-wise methods usually require post-processing with traditional physical models at the final stage. A typical approach is to predict both the wrapped phase (or its equivalent representation) and a coarse absolute phase, which is then refined using physical equations and rounding operations to compensate for small errors and improve point cloud quality [171,172,173]. Recently, such methods have increasingly emphasized explicit integration of FPP physical principles. For example, Jiang et al. [174] proposed a “1-to-6” architecture capable of predicting three pairs of numerators and denominators. It is important to note, however, that although output designs vary, directly predicting fringe order should be avoided, as fringe order is a discrete variable and thus not well suited for regression-based deep learning models.

Overall, supervision strategies play a crucial role in enhancing the robustness and accuracy of fringe-structured-light 3D reconstruction. Deep supervision improves generalization through multi-level regularization, while branch-wise supervision tightly integrates physical priors with network predictions, further improving point cloud quality. Although these approaches differ in mechanisms and computational costs, both demonstrate strong potential to overcome the limitations of conventional end-to-end learning. It is also worth noting that the effectiveness of supervision strategies largely depends on the design of input features. Therefore, the next section will focus on the evolution of input paradigms and their impact on network performance.

#### 5.2.3. Input Design

In FPP systems, the design of input features is of critical importance, as certain features cannot be efficiently learned by neural networks in an automatic manner. In recent years, researchers have sought to overcome this limitation through innovations in input engineering. Nguyen et al. [160] compared various fringe patterns, including speckle patterns, high-frequency fringes, low-frequency fringes, and natural images, and found that high-frequency fringes delivered the best reconstruction performance. However, in traditional Fourier transform-based methods, key parameters that strongly influence accuracy—such as fringe patterns and projection angles—have not yet been fully optimized.

Among different input designs, composite approaches such as color-composite fringes and frequency-composite fringes have demonstrated promising performance. For example, Wang et al. [162] incorporated discrete wavelet transform (DWT) components into the input features and showed that this improved RMSE accuracy by 4% across the entire test dataset. Li et al. [173] employed composite three-frequency fringes as input and simultaneously predicted three intermediate components: unwrapped phase, numerator, and denominator. Their proposed CDLP method outperformed traditional FT approaches, although comparisons with other benchmarks such as sinusoidal fringes remain insufficient. Zhu et al. [175] introduced the SCFPP method, which surpassed CDLP and DCFPP on their self-collected dataset, improving MAE by 20.4%. It is worth noting that while color-composite fringes show potential, their application in measuring colored objects still suffers from limitations and has therefore not been widely adopted.

In summary, further optimization of input feature design remains a key factor for improving both the accuracy and robustness of FPP. The performance variations observed across different fringe patterns and composite approaches not only highlight the importance of input engineering but also point to future research directions. In particular, greater emphasis should be placed on fully exploiting multi-frequency fringe information and integrating multimodal fringe features to further enhance reconstruction accuracy and adaptability.

### 5.3. Evaluation Metrics

In deep learning-driven FPP, designing appropriate evaluation metrics is crucial to ensure the effectiveness and robustness of models in real-world applications. To comprehensively assess model performance, a multi-dimensional evaluation framework that integrates both visualization and quantitative metrics is needed to systematically analyze network behavior across diverse scenarios.

Visual analysis plays a key role in identifying systematic errors and failure modes. In FPP tasks, effective visualization not only intuitively reflects the network’s performance under varying conditions but also reveals limitations in handling specific challenges. However, most existing studies only present phase or point cloud errors in limited scenarios, which cannot fully reflect global performance. Therefore, scene-specific fine-grained testing is recommended to highlight artifacts more clearly and reduce dataset bias.

**Figure 17:**
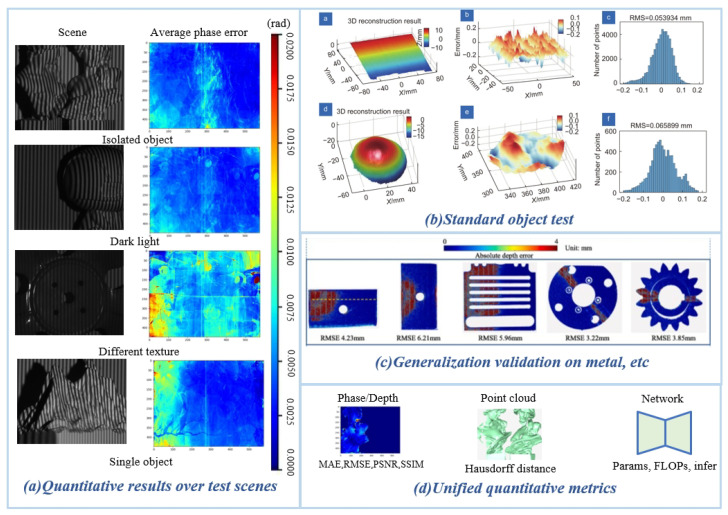
Evaluation metrics for deep learning-enabled FPP. (**a**) Quantitative results over test scenes under different conditions such as isolated object, dark lighting, and textured background. (**b**) Standard object test including 3D reconstruction and error histogram analysis. (**c**) Generalization validation on challenging objects like metallic surfaces. (**d**) Unified quantitative metrics involving phase/depth accuracy, point cloud accuracy, and network complexity.

Figure 17 comprehensively illustrates the evaluation framework of our method, which is organized into four complementary perspectives: quantitative scene evaluation, standard object validation, generalization testing on industrial materials, and unified metric reporting. Per-pixel error heatmaps, as shown in Figure 17a, are a commonly used visualization method. We present quantitative results across diverse test scenes, including multi-object scenarios, isolated targets, low-light conditions, complex textures, and single objects. The left column shows representative fringe or intensity images, while the right column displays average phase error maps (in radians). These heatmaps can clearly present the local error distribution in phase or depth reconstruction, helping to uncover systematic biases. For instance, a model trained on specific objects may generalize poorly to new objects with different textures or reflectance. Taking the FP672 dataset [160], as an example, its data mainly originate from a single statue under uniform lighting conditions. As such, it is insufficient to evaluate the network’s robustness under complex objects and varying illumination. Therefore, it is recommended to include industrially common materials such as metals during the testing phase to more effectively challenge and assess the model’s generalization capabilities.

In addition, non-uniform surface reflectance remains a major challenge, often leading to errors in fringe order prediction. Complex reflective surfaces may hinder the network’s ability to accurately infer fringe orders. To address this issue, it is suggested to augment testing with surfaces that exhibit strong reflectivity, enabling a more realistic assessment of model robustness. In Figure 17c, we validate the model’s generalization capabilities on industrially relevant materials, especially metallic and highly reflective objects. The absolute depth-error maps (unit: mm) highlight the reconstruction challenges posed by strong reflections, while the reported RMSE values under each case quantitatively measure the model’s accuracy in such non-ideal scenarios.

Beyond static scene evaluations, dynamic scene evaluation has gained increasing attention. Temporal consistency visualization offers a valuable extension to traditional static metrics, especially in revealing motion artifacts and the impact of temporal variations [176,177]. Dynamic evaluation is critical for validating model stability and consistency under continuously changing environments, making it particularly applicable to long-term industrial deployments.

To establish a robustness benchmark that more closely reflects real-world scenarios, it is recommended to introduce variable conditions such as background light intensity and changes in ambient illumination. These variations simulate common real-world disturbances (e.g., lighting fluctuations, object occlusion) and enable systematic assessment of the model’s adaptability under non-ideal conditions.

Quantitative metrics provide an objective basis for standardized model performance comparison. However, existing studies lack consensus on metric selection, making direct comparisons across methods difficult. Currently, mean absolute error (MAE) and root mean square error (RMSE) are the most widely adopted basic metrics. In recent years, Peak Signal-to-Noise Ratio (PSNR), Structural Similarity Index Measure (SSIM), and 3σ deviation have also been introduced to characterize prediction errors from different dimensions. These metrics reflect mean error, variance, structural fidelity, and signal-to-noise characteristics, respectively, as shown in Figure 17d. Meanwhile, standard geometric objects, such as spheres and planes, are particularly effective for evaluating the accuracy of the predicted results, as illustrated in Figure 17b. Also, to address the lack of standardization, we propose a unified set of integrated metrics, encompassing phase- and depth-level accuracy (MAE, RMSE, PSNR, SSIM), point-cloud-level fidelity (e.g., Hausdorff distance), and network efficiency (e.g., parameter count, FLOPs, inference time). Therefore, reporting such a comprehensive set of metrics is recommended to ensure more robust and holistic performance evaluation across different approaches.

In FPP systems, quantitative evaluation of point clouds has long been relatively weak. As prediction accuracy improves, relying solely on visual artifacts is no longer sufficient to comprehensively reflect model performance. Thus, it is suggested to incorporate point cloud-specific evaluation metrics such as the Hausdorff distance [178] and Iterative Closest Point (ICP) registration error [179], which accurately measure geometric deviations between predicted and ground-truth point clouds. Moreover, as shown in Figure 17b, standard objects (e.g., spheres and planes) are widely used for systematic comparison across methods due to their geometric simplicity and clearly quantifiable errors, making them valuable benchmarks.

Finally, when reporting overall performance, in addition to traditional accuracy metrics, efficiency-related metrics such as the number of network parameters (Parameters), floating-point operations per second (FLOPs), training time, and inference time should also be included. It is worth noting that parameter count and FLOPs do not always directly correlate with inference speed. Therefore, it is recommended to comprehensively report all relevant metrics to provide a more complete basis for evaluating the trade-offs between accuracy and efficiency.

## 6. Challenges and Perspectives

In the development of fringe-structured-light systems, addressing the challenges of reconstruction under complex environments and dynamic scenes has always been a central research focus. In recent years, as application demands continue to expand, fringe-structured-light systems are required not only to maintain measurement accuracy in high dynamic range (HDR) scenarios but also to achieve stable reconstruction under conditions such as limited depth of field and rapid target motion [179,180,181,182]. Meanwhile, real-time monitoring capabilities have become increasingly essential in fields like intelligent manufacturing and industrial inspection. As deep learning emerges as a key tool for enhancing system performance, issues related to its interpretability and transferability have also attracted growing attention.

### 6.1. HDR Issues

In complex surface 3D measurement tasks, high dynamic range (HDR) imaging poses a significant challenge. Highly reflective regions are prone to image saturation, while low-reflectivity areas may suffer from low signal-to-noise ratios (SNR), leading to unstable phase estimation and, consequently, degraded 3D reconstruction accuracy. Traditional FPP systems often struggle to achieve ideal imaging quality across all regions under such conditions using a single fixed exposure setting [183].

In recent years, deep learning has offered new solutions for 3D measurement under HDR conditions. Zhang et al. were the first to introduce deep neural networks into HDR 3D reconstruction, using the results of a 12-step phase-shifting method as supervision to train a 3-step model, thereby increasing the dynamic range by 4.8 times [184]. However, the 12-step images still suffer from saturation in highly reflective regions, limiting their validity as ground truth. Subsequent studies have shown that deep models can learn the mapping between fringe patterns and phase, significantly reducing the number of required projections and enabling fast reconstruction under HDR conditions [184,185,186,187].

Nevertheless, deep learning models are highly sensitive to the distribution of training data, and publicly available HDR 3D reconstruction datasets remain extremely scarce. For example, Y-FFCNet, trained on simulated data, achieved separation of specular and diffuse reflections in highly reflective regions, significantly improving reconstruction performance for metallic objects [188]. However, accurate decoupling of reflection components remains challenging, and the generalization gap caused by synthetic data has yet to be resolved. Liu et al. proposed the SP-CAN method, which simulates a multi-exposure process using a neural network to enhance feature reconstruction in HDR regions [189]. However, its reliance on low-exposure fringe images may lead to insufficient feature representation, and the optimal exposure time still requires manual tuning.

In summary, future research should focus on building HDR 3D measurement datasets that better reflect real-world scenarios to improve model robustness to illumination and reflectivity variations. Moreover, the development of self-supervised or weakly supervised learning strategies can help reduce reliance on high-quality ground truth data. Lastly, designing network architectures with improved interpretability and generalizability will be essential to promote the practical deployment of HDR 3D reconstruction technologies.

### 6.2. Extended Depth of Field

In structured-light 3D measurement systems, extending the depth of field (DOF) is a key research direction for enhancing system adaptability and reconstruction accuracy. In practical scenarios, variations in object height often exceed the system’s focal range, leading to severely blurred regions that cause phase errors and reconstruction deviations—commonly referred to as the “local blur problem” [190].

To tackle the challenges posed by limited DOF, traditional approaches have proposed a variety of strategies to improve robustness. For instance, Drouin et al. [191] introduced an iterative deconvolution-based pattern segmentation method that enhances image sharpness to detect blurred edges. In their subsequent work [192], they estimated spatially varying point spread functions (PSFs) by projecting dot patterns within a calibrated measurement volume, enabling more accurate modeling and compensation of blur effects. Chen et al. [193] proposed a technique that combines polarization with high-frequency fringe patterns to reduce errors caused by subsurface scattering, and further developed the Modulated PS method [194], which enables 3D reconstruction without explicitly separating direct and indirect light components. Additional strategies, such as MicroPS [195], unstructured-light techniques [196], and embedded phase-shifting methods [197], aim to suppress the influence of indirect light through high-frequency encoding, thereby enhancing the system’s ability to manage blurred regions.

While these methods have mitigated defocus-related issues to some extent, they largely remain within the realm of traditional image processing paradigms, relying heavily on pattern design and physical modeling. They have yet to fully exploit the powerful feature extraction and nonlinear modeling capabilities offered by deep learning. Future research should explore the integration of deep neural networks to build end-to-end frameworks for blur region detection and error correction. In particular, a jointly optimized approach that combines high-frequency pattern design, PSF estimation in blurred areas, and end-to-end phase error correction could substantially improve the robustness and accuracy of structured-light systems under conditions of large depth-of-field and complex surface geometries.

### 6.3. High-Speed Deployment and Real-Time Reconstruction

In high-speed dynamic or transient measurement scenarios, the performance of FPP is constrained by the refresh rates of projection and acquisition hardware, as well as the computational efficiency of reconstruction algorithms [198]. These limitations significantly hinder its ability to meet the demands of real-time 3D reconstruction tasks that require high speed, high accuracy, and low latency. Traditional FPP methods typically rely on capturing multiple 8-bit sinusoidal fringe patterns to extract absolute phase information. However, the system’s frame rate is often limited by the flipping speed of digital micromirror devices and the camera’s exposure time, making high-frame-rate operation difficult to achieve [199,200].

To overcome these constraints, researchers have proposed the binary defocusing technique, which generates quasi-sinusoidal fringe patterns by projecting slightly defocused 1-bit binary images [201,202,203]. This approach fully leverages the high-speed switching capability of DMDs, enabling fringe projection rates in the kilohertz range. Moreover, by reducing the imaging window, high-speed cameras can achieve acquisition rates of up to 100,000 frames per second. When combined with deep learning methods—such as image super-resolution and single-frame phase decoding—this enables ultra-fast single-frame 3D imaging, as demonstrated in the SSSR-FPP method [204]. This line of research indicates that deep learning has great potential to significantly accelerate imaging speed without compromising measurement accuracy.

Despite these promising advances, deep learning-integrated FPP systems still face several challenges in practical deployment. Future research should focus on lightweight network architecture design, platform-aware optimization strategies, and multi-task end-to-end integration. Additionally, system-level co-design of hardware and software will be essential for developing real-time 3D reconstruction systems that are high in accuracy, low in latency, and energy-efficient for real-world applications.

### 6.4. Transferability, Generalization, and Interpretability of Deep Learning Methods

Although traditional structured-light systems offer strong customization and high measurement accuracy, they often rely on fixed configuration parameters, limiting their adaptability across different devices and environments [205,206]. This limitation has driven researchers to explore transfer learning strategies [207] to bridge the gap between simulation and reality and to achieve cross-configuration generalization. Currently, such methods are primarily applied in speckle-based structured-light systems or downstream tasks like eye tracking [208], while their application in line-structured-light 3D measurement remains underexplored.

In recent years, the emergence of large-scale models has significantly improved generalization and transfer capabilities across various fields, providing new opportunities for the intelligent development of FPP systems. By introducing foundation models or developing domain-adaptive fine-tuning mechanisms tailored to FPP data characteristics, future systems are expected to exhibit enhanced robustness and accuracy in unseen scenarios while reducing the need for repeated task-specific training, thereby enabling more efficient cross-task adaptation.

At the same time, deep learning has reshaped the development landscape of single-frame FPP systems, often surpassing traditional methods in terms of speed, accuracy, and robustness—particularly in dynamic scenes and complex surfaces. However, the physical mechanisms underlying these advantages remain poorly understood, and deep networks are still largely treated as “black boxes.” As a result, improving the interpretability of deep models has become a research priority. Some studies have explored methods such as feature map visualization [209] to reveal how networks extract and process complex fringe patterns, aiming to shift from “black box” to “gray box” modeling and improve transparency. Nevertheless, systematic investigations into interpretability methods within FPP systems remain limited, and their impact on model reliability, tunability, and generalization performance still demands further exploration.

Future research should focus on building diverse, high-quality cross-domain FPP datasets and introducing few-shot learning and domain adaptation strategies to improve transferability and generalization. In parallel, efforts to enhance model interpretability—such as visualizing internal features—are essential for uncovering model mechanisms and improving system transparency, reliability, and controllability.

## 7. Conclusions

As application demands continue to grow, structured-light 3D reconstruction systems are evolving toward higher precision, greater portability, enhanced intelligence, and stronger robustness. This paper provides a systematic comparison between the two mainstream methods, namely FPP and PMD, from the perspective of system architecture and fundamental principles. It highlights their differences and complementary advantages in terms of measurement mechanisms, applicable surface types, modeling strategies, and error control approaches. At the same time, MEMS-based micromirror scanning technology is becoming a promising direction for next-generation structured-light systems because of its lens-free configuration, large depth of field, compact structure, and high-speed operation. For system calibration, unified models, isophase surface models, and phase-angle models specifically developed for MEMS systems provide effective tools for modeling nonlinear optical paths. To mitigate issues such as projection errors, light source fluctuations, and high-order harmonic distortions, researchers have introduced various compensation strategies at both the hardware and algorithmic levels, significantly improving the robustness of the overall system.

The integration of deep learning has introduced a paradigm shift in structured-light measurement. Whether for single-frame reconstruction or multi-frame nonlinear mapping, deep neural networks consistently outperform traditional algorithms. Advances in network architecture, incorporation of physical priors, input feature engineering, and evaluation metric design have opened new paths for accurate reconstruction under complex scenes.

Looking ahead, structured-light 3D reconstruction still faces several challenges and opportunities. At the hardware level, improvements are required in the precision, power stability, and cost efficiency of MEMS projectors. At the modeling level, it is important to integrate geometric priors, optical imperfections, and learning-based approaches to enhance system adaptability across different platforms and complex environments. At the intelligence level, further exploration of deep learning techniques is needed in areas such as few-shot learning, weak supervision, and multimodal fusion, with a particular focus on developing end-to-end models that incorporate physical constraints.

Moreover, to facilitate the practical deployment of structured-light systems in industrial and service settings, building generalized and portable evaluation datasets and performance metrics will be a crucial step. In summary, structured-light 3D reconstruction is undergoing a pivotal transformation through the deep integration of traditional methods and intelligent technologies, and is expected to play an increasingly important role in fields such as precision manufacturing, soft robotics, cultural preservation, and intelligent interaction. We believe this review can serve as a valuable reference for researchers and engineers, providing both a clear understanding of current advances and a forward-looking perspective on future development in the field.

## Figures and Tables

**Table 1:** Representative reviews and studies on fringe-based structured-light 3D reconstruction.

Author	Year	FPP	PMD	MEMS	Deep Learning	Description
Tobias Möller et al. [33]	2005	×	✓	×	×	Early review of PMD-range imaging
Xu et al. [34]	2020	×	✓	×	×	PMD for 3D specular-surface measurement
Lv et al. [36]	2020	✓	×	×	×	FPP measurement theory
Kulkarni et al. [38]	2020	✓	×	✓	×	Fringe denoising algorithms
He et al. [35]	2021	✓	×	×	×	Temporal-phase unwrapping methods
Liu et al. [39]	2024	✓	×	×	✓	Deep learning in fringe projection
Bai et al. [37]	2024	✓	✓	×	✓	Three-dimensional shape measurement
Our article	2025	✓	✓	✓	✓	First comprehensive review systematically summarizing FPP, PMD, MEMS, and deep learning integration

**Table 2:** Performance comparison of typical structured-light systems.

Parameter	Interference	Physical Grating	LCD	DLP	MEMS
Accuracy	$10^{-1}$ mm	$10^{-3}$ mm	$10^{-2}$ mm	$10^{-3}$ mm	$10^{-3}$ mm
Speed	∼50 fps	∼100 fps	∼50 fps	∼120 fps	>1000 fps
Resolution	<1 K	<1 K	∼1 K	∼1 K	>4 K
Programmable	No	No	Yes	Yes	Yes
Power Consumption	∼100 W	∼300 W	∼40 W	∼50 W	∼5 W
Cost	>$10,000	>$10,000	∼$1500	∼$2000	∼$500
Optical Efficiency	Medium	Low	Medium	Low	High

## Data Availability

No new data were created or analyzed in this study. Data sharing is not applicable to this article.

## Abbreviations

The following abbreviations are used in this manuscript:

FPP	Fringe Projection Profilometry
PMD	Phase Measuring Deflectometry
DLP	Digital Light Processing
TPU	Temporal-Phase Unwrapping
SPU	Spatial-Phase Unwrapping
LCD	Liquid-Crystal Display
DMD	Digital Micromirror Device
MEMS	Micro-Electro-Mechanical Systems
CMM	Coordinate Measuring Machines
SOTA	State-of-the-Art
CNNs	Convolutional Neural Networks
MAE	Mean Absolute Error
MSE	Mean Squared Error
RMSE	Root Mean Square Error
PSNR	Peak Signal-to-Noise Ratio
SSIM	Structural Similarity Index Measure
HDR	High Dynamic Range
SNR	Signal-to-Noise Ratios
DOF	Depth of Field
PSF	Point Spread Function
